# Systematic screening reveals synergistic interactions that overcome MAPK inhibitor resistance in cancer cells

**DOI:** 10.20892/j.issn.2095-3941.2020.0560

**Published:** 2021-06-09

**Authors:** Yu Yu, Minzhen Tao, Libin Xu, Lei Cao, Baoyu Le, Na An, Jilin Dong, Yajie Xu, Baoxing Yang, Wei Li, Bing Liu, Qiong Wu, Yinying Lu, Zhen Xie, Xiaohua Lian

**Affiliations:** 1 Department of Cell Biology, Basic Medical College, Army Medical University (Third Military Medical University), Chongqing 400038, China; 2 MOE Key Laboratory of Bioinformatics and Bioinformatics Division, Center for Synthetic and System Biology, Department of Automation, Beijing National Research Center for Information Science and Technology, Tsinghua University, Beijing 100084, China; 3 National Cancer Center/National Clinical Research Center for Cancer/Cancer Hospital, Chinese Academy of Medical Sciences and Peking Union Medical College, Beijing 100021, China; 4 Department of Thoracic Surgery, Peking Union Medical College Hospital, Chinese Academy of Medical Sciences, Beijing 100730, China; 5 Beijing Syngentech Co., Ltd, Beijing 102206, China; 6 School of Life Sciences, Tsinghua University, Beijing 100084, China; 7 The Comprehensive Liver Cancer Center, The 5th Medical Center of PLA General Hospital, Beijing 100039, China

**Keywords:** Pair-wise sgRNA library, genetic interactions, MAPKi resistance, combinatorial therapy, cancer stemness

## Abstract

**Objective:**

Effective adjuvant therapeutic strategies are urgently needed to overcome MAPK inhibitor (MAPKi) resistance, which is one of the most common forms of resistance that has emerged in many types of cancers. Here, we aimed to systematically identify the genetic interactions underlying MAPKi resistance, and to further investigate the mechanisms that produce the genetic interactions that generate synergistic MAPKi resistance.

**Methods:**

We conducted a comprehensive pair-wise sgRNA-based high-throughput screening assay to identify synergistic interactions that sensitized cancer cells to MAPKi, and validated 3 genetic combinations through competitive growth, cell viability, and spheroid formation assays. We next conducted Kaplan-Meier survival analysis based on The Cancer Genome Atlas database and conducted immunohistochemistry to determine the clinical relevance of these synergistic combinations. We also investigated the MAPKi resistance mechanisms of these validated synergistic combinations by using co-immunoprecipitation, Western blot, qRT-PCR, and immunofluorescence assays.

**Results:**

We constructed a systematic interaction network of MAPKi resistance and identified 3 novel synergistic combinations that effectively targeted MAPKi resistance (*ITGB3* + *IGF1R, ITGB3* + *JNK*, and *HDGF* + *LGR5*). We next analyzed their clinical relevance and the mechanisms by which they sensitized cancer cells to MAPKi exposure. Specifically, we discovered a novel protein complex, HDGF-LGR5, that adaptively responded to MAPKi to enhance cancer cell stemness, which was up- or downregulated by the inhibitors of ITGB3 + JNK or ITGB3 + IGF1R.

**Conclusions:**

Pair-wise sgRNA library screening provided systematic insights into elucidating MAPKi resistance in cancer cells. ITGB3-+ IGF1R-targeting drugs (cilengitide + linsitinib) could be used as an effective therapy for suppressing the adaptive formation of the HDGF-LGR5 protein complex, which enhanced cancer stemness during MAPKi stress.

## Introduction

Aberrant activation of the mitogen-activated protein kinase (MAPK)/extracellular signal-regulated kinase (ERK) pathway is common in many cancer species. In melanomas, the mutation of valine to glutamic acid at amino acid 600 in BRAF (BRAF^V600E^) occurs in more than 50% of patients, and can boost kinase activity and constitutively promote the activation of MEK/ERK signaling, which endows cancer cells with increased proliferation, survival, and metastasis^1–4^. Although MAPK inhibitors (MAPKi) such as vemurafenib (PLX4032), dabrafenib, and trametinib have been approved as clinical drugs for late-stage tumors, most patients ultimately develop lethal resistance to MAPKi treatment^5^. Understanding the molecular mechanism of MAPKi resistance is therefore essential to improving the efficacy of MAPKi treatment and preventing disease relapse.

To systematically analyze potential MAPKi resistance mechanisms, we selected A375 melanoma cells with typical MAPK hyperactivation characteristics as our high-throughput screening model. We included regulators that influenced several critical biological and cellular processes that have been shown to be involved in bypassing the BRAF^V600E^ inhibition in melanoma cells; for example, modulators of MAPK, GTPases, and epigenetic modifications; regulators of cell proliferation and cell death; facilitators of the epithelial-mesenchymal transition (EMT); and factors that sustain cancer stem cell maintenance^6–27^. Additionally, the STAGA and MEDIATOR complexes, which regulate transcription from RNA polymerase II promoters, are essential for vemurafenib resistance, possibly due to their contributions to excessive metabolism or repression of cell apoptosis^28–30^. However, combinations of multiple resistance mechanisms may occur in melanoma cells, and further studies are needed to elucidate the functional relationships between individual genes involved in MAPKi resistance^31,32^.

The functional relationships between 2 genes, also termed genetic interaction (GI), can be evaluated by comparing the phenotypes of cells with simultaneous mutations in 2 genes to the phenotypes of cells with a single mutation^33^. Systematic identification of genetic interactions in mammalian cells is first facilitated by using pooled screening or large-scale automated transfection, in which 2 small hairpin RNAs (shRNAs) are introduced into individual cells to simultaneously perturb 2 genes^34,35^. More recently, a multiplexed CRISPR/Cas9 screening approach has been developed by using a library of barcoded pair-wise single-guide RNA (sgRNA) combinations to search for potential growth-inhibiting gene pairs in cancer cells, which can facilitate the development of combinatorial cancer therapy^36–39^. Furthermore, concurrent suppression of BRAF, MEK, and ERK has been used to prevent the propagation of cells with high level amplification of *BRAF* mutations, highlighting a practical strategy for overcoming vemurafenib resistance^40^. However, until now, these studies have been restricted to limited gene combinations, and have been focused mainly on MAPK signaling itself and its best known bypassing pathways, such as PI3K^41^. They therefore lack comprehensive insight into the cooperative mechanisms responsible for the occurrences of effective gene pairs.

Elevated signals related to receptor tyrosine kinases (RTKs)^17^, the EMT, and cancer stem cells (CSCs)^42^ have emerged as 3 major causes of drug resistance in diverse species of cancer cells receiving various kinds of therapies. However, most studies have focused on only 1 of these regulatory processes^43,44^. How these kinds of signals interact with one another to generate adaptive resistance in refractory cancer cells remains poorly understood. In this study, we aimed to identify genetic interactions among 84 genes reported to be involved in the RTK-, EMT-, and CSC-induced drug resistances of cancer cells, to identify potent combinatorial regimens that are universally applicable in sensitizing a wide variety of cancer cells.

To identify the genetic interactions among RTK-, EMT-, and CSC-relevant genes, we developed an assembly method to efficiently construct a library of more than 40,000 pair-wise sgRNA combinations. We then performed pooled CRISPR/Cas9 screening in cultured human A375 melanoma cells that contained the BRAF^V600E^ mutation. Next, we evaluated the genetic interaction of each gene pair by calculating the phenotypic value of vemurafenib resistance, validated 3 identified novel gene pairs with strong *GI* scores by using a cell growth competition assay and small molecule inhibition assay, and experimentally identified the underlying synergetic mechanisms of multidrug regimens. These results provided comprehensive information about potential critical connections between essential genes that may influence vemurafenib resistance in melanoma cells. The methods developed in this study enabled the efficient assembly of pair-wise sgRNA libraries and facilitated systematic analyses of genetic interaction networks of complex biological processes.

To deliver an adjuvant sensitizing effect to MAPKi resistance in an expanded panel of cancer cell lines with hyperactivated MAPK signaling, we also tested our adjuvant sensitizing pairs across cell lines within multiple cancer species, including melanoma (MeWo), hepatocarcinoma (MHCC97H), lung adenocarcinoma (H1299), and breast cancer (MCF7) cells.

By analyzing clinical samples from melanoma patients with different grades of stemness, we noted that HDGF and LGR5 correlated with the expression of a commonly used CSC marker, CD133, which was co-localized in the microvascular regions of melanoma tissues. Next, using cell line models, we found that the simultaneous suppression of *HDGF* and *LGR5* synergistically disrupted the formation of cancer spheroids and downregulated the level of cancer stem cell markers. Consistently, we found that they paired as a physical complex that allowed cancer cells to adapt to the vemurafenib/dabrafenib/trametinib environment. To the best of our knowledge, this is the first study to identify and analyze a MAPKi stress-responsive protein complex that adaptively induced resistance. We also provided a detailed study of the coordination of *HDGF-LGR5* in achieving adaptive drug resistance by sustaining EMT signals and CSC maintenance. These observations suggested that *HDGF* and *LGR5* acted in concert to affect the stemness and survival of BRAFi (BRAF inhibitor)-treated cancer cells, which is worth developing as a future therapy.

## Materials and methods

### Patients

Melanoma patient specimens (No. 882775, No. 848557, No. 842601, and No. 844583) used for immunohistochemistry (IHC) and immunofluorescence (IF) staining were collected at the National Cancer Center of Chinese Academy of Medical Sciences and Peking Union Medical College, after informed written consent was obtained from each patient or each patient’s guardian according to the institutional guidelines and the principles of the Declaration of Helsinki. Tumor tissue sections were stained with anti-ITGB3, anti-IGF1R, or anti-phospho-JNK antibodies for IHC analyses, and anti-CD133 + anti-BMI1, anti-HDGF + anti-LGR5 antibodies for IF analyses.

### Cell culture

The HEK293 (293-FT) cell line was purchased from Life Technologies (Carlsbad, CA, USA). The A375 cell line was obtained from the National Infrastructure of Cell Line Resource (Beijing, China), MeWo cells were purchased from Fenghbio (Beijing, China), H1299 and MCF7 cells were acquired from Procell Life Science & Technology (Waltham, MA, USA), and MHCC97H cells were purchased from Beyotime Biotechnology (Jiangsu, China). No cell line used in this study was found in the database of commonly misidentified cell lines maintained by ICLAC (https://iclac.org/) and NCBI Biosample (https://www.ncbi.nlm.nih.gov/biosample). The cell lines tested negative for mycoplasma contamination and were regularly treated with mycoplasma removing agent. TransStble3 and Trans5 α competent cells were purchased from TransGen Biotech (Beijing, China).

### Reagents and enzymes

Q5 High-Fidelity DNA polymerase, T4 DNA ligase, and restriction endonucleases of BsaI and Esp3I were purchased from New England Biolabs (Ipswich, MA, USA). Polyethylenimine (PEI) was purchased from Polysciences (Warrington, PA, USA). Polybrene and puromycin were purchased from Sigma-Aldrich (St. Louis, MO, USA). Hoechst/propidium iodide (PI) double staining system, kanamycin, and spectinomycin were obtained from Solarbio (Beijing, China). Mycoplasma removing agent was purchased from Solarbio. Vemurafenib, cilengitide, linsitinib, and JNK-IN-8 were acquired from Selleck (Houston, TX, USA). TRIzol and SYBR Green PCR Master Mix were purchased from Beyotime Biotechnology and Thermal Fisher Scientific (Waltham, MA, USA). Primary antibody to BIRC5 was purchased from Solarbio. Primary antibodies to phospho-ERK1^Thr202/Tyr204^/ERK2^Thr185/Tyr187^, cleaved caspase-3, β-catenin, phospho-FAK, BMI1, OCT4, nestin, p75 NGFR, CD133, β-actin, and secondary antibodies conjugated to horseradish peroxidase were purchased from Beyotime Biotechnology. Primary antibody to HDGF was purchased from Proteintech (Rosemont, IL, USA) and primary antibody to LGR5 was obtained from Sino Biological (Beijing, China).

### Efficiency test of 2 promoters in the pair-wise sgRNA vector

The coding region of the fluorescence protein of mKate or EYFP was split into 2 fragments, and a designed linker that was recognized by the CRISPR/Cas9 system was added between the fragments (**Supplementary Figure S1A and S1C**). The promoters of U6 and 7SK were then assembled into the vectors, which are listed (the detailed sequence information of sgRNA is shown in **Supplementary Table S1**).

U6-mKate_linker_sgRNA-7SK-negative_sgRNA-EF1α-spCas9;

U6-negative_sgRNA-7SK-mKate_linker_sgRNA-EF1α-spCas9;

U6-mKate_linker_sgRNA-7SK-EYFP_linker_sgRNA-EF1α-spCas9;

U6-EYFP_linker_sgRNA-7SK-mKate_linker_sgRNA-EF1α-spCas9;

U6-negative_sgRNA-7SK-negative_sgRNA-EF1α-spCas9;

Next, the PEI induced transfection assays were conducted by combining the plasmid carrying expression fragments of mKate with either of the first 2 vectors as well as the negative control vector V packaged by using PEI at the ratio of 1:3 for the single activation test. Additionally, the plasmids carrying expression fragments of mKate and EYFP were combined with vectors III, IV, or V above, and the same method was separately used for double activation testing. These transfection mixtures were added into 24-well plates of 293FT cells using 3 experimental replicates.

After 2 days, the cells were harvested and analyzed by fluorescence-activated cell sorting. Subsequently, the percentages of mKate in the experimental groups of vectors I and II or the percentages of mKate and EYFP in the experimental groups of vectors III and IV were divided by the corresponding percentage of leaky fluorescence in the negative control groups of vector V, which was then defined as the relative fluorescence expression level. Finally, we used the 2-tailed unpaired *t*-test to evaluate the statistical significance of the relative fluorescence activated by different vectors with adversely located promoters of U6 and 7SK.

### Construction of plasmid DNA and sgRNA libraries

The vectors used in this study were constructed using standard molecular cloning techniques, including restriction enzyme digestion, ligation, PCR, and Golden Gate Assembly. Custom oligonucleotides were purchased from Genewiz (Beijing, China). The vector constructs were transformed into *E. coli* strain, Trans5α, and 50 μg/mL of kanamycin/spectinomycin was used to isolate colonies harboring the constructs. DNA was then extracted and purified. Sequences of the vector constructs were verified by DNA sequencing.

We synthesized a control oligo pool for constructing 12 negative control sgRNAs, and 3 oligo pools for constructing sgRNAs. Each oligo pool included 84 sgRNA spacers targeting 84 genes. To construct the sgRNA vector containing the U6 promoter (U6p) or 7SK promotor (7SKp)-driven expression of a sgRNA that targeted a specific gene, the 20 bp sgRNA spacer oligos were annealed and cloned into 7SK-pHS-BVC-LW002 or U6-pHS-BVC-LW003 vectors by using BsaI. To generate the pooled sgRNA vector sublibraries, 84 annealed sgRNA spacer oligos in each pool were mixed with 12 annealed negative control sgRNA spacer oligos and the annealed S1:S2 spacer oligos (S1 sequence: 5′-ACCGATCCTCACGGTGAACGTCT-3′; S2 sequence: 5′-AAACAGACGTTCACCGTGAGGAT-3′) with equal molar ratios and cloned them into the 7SK-pHS-BVC-LW002 or U6-pHS-BVC-LW003 vectors in a Golden Gate reaction by using BsaI, generating single-wise sgRNA libraries (7SK-pHS-BVC-LW002-library and U6-pHS-BVC-LW003-library). The destination pair-wise sgRNA lentiviral vector (pHS-BGL-YR001) and single-wise sgRNA libraries (7SK-pHS-BVC-LW002-library and U6-pHS-BVC-LW003-library) were mixed at equal molar ratios in the second round of the Golden-Gate reaction by using Esp3I, resulting in a pair-wise sgRNA library. The pair-wise sgRNA library plasmid DNAs were prepared by using TransStble3 competent cells, and then purified.

### Lentivirus production and library screening

Pooled lentiviral library plasmid DNA was a mixture with equal molar ratios of 6 pair-wise sgRNA libraries, 3 single-wise sgRNA libraries, and a negative control library. Pooled lentiviruses were produced and packaged as described with minor modifications. Briefly, 20 μg of pooled lentiviral library plasmid DNA, 20 μg of pCMV-dR8.2-dvpr, and 15 μg of pCMV-VSV-G were transfected into HEK293FT cells in a 15 cm petri dish by using PEI transfection reagents. Approximately 1 × 10^8^ Cas9-A375 cells were infected with pooled sgRNA libraries at 0.3 MOI to maintain library coverage at approximately 1,000-fold. One day after infection, the cells were selected with 1 mg/mL puromycin prior to further experiments. Selected A375 cells were then treated with vemurafenib or dimethyl sulfoxide (DMSO) for 10 days. The A375 genomic DNA was extracted from harvested A375 cells. Approximately 569 bp DNA fragments containing the pair-wise sgRNA region were amplified by PCR with 25 thermal cycles from A375 genomic DNA using primers (5′-ATTTGTCTCGAGGTCGAGAATTC-3′ and 5′-TCATATGCTTACCGTAACTTG-3′) using Q5 high-fidelity DNA polymerase. The DNA fragments were used to construct DNA libraries (BGI, Yantien, China) by combining the fragmented DNA and end repair mix (BGI), then purifying them before conducting the A-tailing reaction to obtain the product of the adenylate 3′-end DNA. PCR free index adapters, (BGI) and the T4 DNA ligase mix, were added to the A-tailing product for ligation of adapters. Finally, adapter-ligated DNA samples were purified for deep sequencing using HiSeq 2500 (Illumina, San Diego, CA, USA) with the 250 nucleotide paired-end mode.

### Sequencing data processing

The quality of deep sequencing data was first evaluated by using FastQC [Babraham Bioinformatics (https://www.bioinformatics.babraham.ac.uk/projects/fastqc/)]. The paired-end sequencing reads were mapped to designed sequences of sgRNA expressing library constructs, where an exact match was required in the seed region of sgRNA, while two mismatches were allowed elsewhere. The paired-end reads that contained duplicates of 2 identical promoters and harbored dislocated sgRNAs were eliminated in the subsequent analysis. The numbers of paired-end reads for each unique pair-wise sgRNA combination in a given sample were normalized as follows:

$$\begin{array}{l} Normalized\;reads\;per\;combination\\ =\frac{Reads\;per\;combination}{Total\;reads\;for\;all\;combinations\;inthe\;sample\times 1.6\times 10^{7}+0.05} \end{array}$$

where 1.6 × 10^7^ represented the maximal total reads among all samples, and 0.05 was added to facilitate calculating phenotypic values in a logarithm scale.

To quantify the growth phenotypes of Cas9-A375 cells with different sgRNA perturbations, we defined the ρ score, which was adapted from a previous study^34^ as follows:

$$\begin{array}{l} \rho_{A}=\frac{1}{kt}\times \log_{2}\left(\frac{N_{A}^{PLX}/N_{WT}^{PLX}}{N_{A}^{DMSO}/N_{WT}^{DMSO}}\right)\\ \rho_{B}=\frac{1}{kt}\times \log_{2}\left(\frac{N_{B}^{PLX}/N_{WT}^{PLX}}{N_{B}^{DMSO}/N_{WT}^{DMSO}}\right)\\ \rho_{AB}=\frac{1}{kt}\times \log_{2}\left(\frac{N_{AB}^{PLX}/N_{WT}^{PLX}}{N_{AB}^{DMSO}/N_{WT}^{DMSO}}\right), \end{array}$$

where *k* was the cell doubling difference of wild-type cells cultured between vemurafenib and DMSO, and *t* denoted the cultured time duration. *N_X_* was the normalized read count of sgRNA combinations targeting single genes (A or B) and gene pairs (AB), while *N_WT_* represented the median read counts of the negative pair-wise sgRNA control in DMSO or the vemurafenib sample. After calculating the ρ score for each pair-wise sgRNA combination, we averaged the sgRNA combinations targeting the same gene or gene pair and excluded the minimal and maximal scores to obtain the quantitative growth phenotype for the corresponding single- or pair-wise perturbation. The value of *P* for a given gene pair was calculated by comparing the distribution of ρ scores targeting the pair of interest to the negative control using the Mann-Whitney U test.

To calculate the genetic interaction strength from the phenotypic ρ score of high-throughput assays, we defined *GI* scores as the deviation of the observed phenotype (ρ_observed_) from the expected phenotype (ρ_expected_) calculated by linear regression analysis using the R package (The R Project for Statistical Computing, Vienna, Austria). The *GI* and ρ scores below the 10th percentile and above the 90th percentile were selected to construct the edge-weighted hierarchical network. Network hubs were identified by using Eigenvector.

To define the synergistic or antagonistic effect of gene combinations, the genetic interaction strength was calculated using Loewe’s additivity principle (*GI*_additivity_ = |pair-wise phenotypic ρ score|−|single-wise phenotypic ρ score 1 + single-wise phenotypic ρ score 2|)^45^.

### Kaplan-Meier survival curve plots

The BRAF mutant cohort from The Cancer Genome Atlas (TCGA) database was selected, and the RNA expression levels of *NF2*, *ITGB3*, and *IGF1R* from the patients with mutated BRAF were discretized into 5 classes as “higher”, “high”, “medium”, “low”, or “lower” using the BurStMisc::ntile() function. The lower expressions of “ITGB3” and “IGF1R” in the figure were defined as patients with *ITGB3* and *IGF1R* expressions below the “low” level. The high expression of *NF2* in the figure was defined as patients with *NF2* expressions above the “medium” level.

### Cell growth competition assays

Cas9-A375 cell lines with sgRNA perturbations of either the single or double gene of 3 gene pairs (*ITGB3 + IGF1R*, and *NF2 + CCNC*) were made using the lentivirus-mediated integration system as previously mentioned. The sgRNA sequences used are listed in **Supplementary Table S2**. For each gene pair, 2 × 10^4^ cells of individual cell lines for a single- or pair-wise gene perturbation were mixed and cultured in DMEM media plus 10% fetal bovine serum (FBS). One day after cell seeding, the cells were split into 2 halves and treated with 2 μM vemurafenib or DMSO in equal volumes for up to 12 days. FACS analysis was conducted using LSRII Fortessa (Becton Dickinson, Franklin Lakes, NJ, USA) as previously described^46^. At least 2 × 10^4^ cell events were acquired per sample. FACS data were analyzed using FlowJo software (https://www.flowjo.com/).

### Small molecule inhibition assays

Small molecule inhibitors (cilengitide for ITGB3, linsitinib for IGF1R, and JNK-IN-8) were used in the assays. Approximately 1 × 10^4^ A375 cells were seeded in a 96-well plate 1 day before the inhibition assay with 2 drug combinations of cilengitide and linsitinib, or cilengitide and JNK-IN-8. For each combinatorial drug treatment, A375 cells were treated with either 2 μM vemurafenib or DMSO in equal volumes, plus individual drugs or drug combinations for 2 days. To label live and dead cells after drug treatments, 100 μL of Hoechst 33342/PI mixed solution was added into each well of the 96-well plate and incubated at 4 °C for 20 min. After washing with 100 μL phosphate-buffered saline (PBS), A375 cells were measured using the high content cellular screening system using UV/488 nm dual excitation and emission wavelengths at 460 nm for Hoechst 33342 and 575 nm for PI. The number of live cells (referred to as *N*) was calculated as follows: *N* = total cell counts - high blue fluorescence counts - high red fluorescence counts. The ρ scores were calculated using the formula as previously described.

### Quantitative RT-PCR

Total RNAs were extracted from A375 cells using TRIzol (Invitrogen, Carlsbad, CA, USA), and the amounts of RNA from different samples were measured using a Nanodrop2000 (Thermo Fisher Scientific). Then, 1.5 μg RNA of the samples was diluted into a whole RNA amplification mixture containing oligo(dT) and dNTPs. A reverse transcription mixture containing reverse transcriptase, dithiothreitol, and first strand buffer were added to the amplified RNA mixture to synthesize cDNA. Next, SYBR Green was added with primers for the E-cadherin, vimentin, and β*-*actin loading control into the cDNA template (at a final dilution of 1:10). Finally, the expression-fold change was calculated using 2^−ΔΔCT^, where ΔΔCT = ΔCT (gene of interest) − ΔCT(loading control) (**Table 1**).

**Table 1:** The primer sequences for qPCR amplification of E-cadherin, vimentin and β-actin

Primer name	Sequence
E-cadherin-F	5′-AAAGGCCCATTTCCTAAAAACCT-3′
E-cadherin-R	5′-TGCGTTCTCTATCCAGAGGCT-3′
Vimentin-F	5′-GACGCCATCAACACCGAGTT-3′
Vimentin-R	5′-CTTTGTCGTTGGTTAGCTGGT-3′
β-actin-F	5′-CATGTACGTTGCTATCCAGGC-3′
β-actin-R	5′-CTCCTTAATGTCACGCACGAT-3′

### Western blot assays

Cell lysates were extracted by using RIPA lysis buffer (Beyotime Biotechnology), and the protein concentrations of different samples were determined using the BCA Protein Assay Kit (Beyotime Biotechnology). A total of 25 μg of each protein sample was separated by SDS-PAGE and transferred to 0.45 μm polyvinylidene difluoride membranes (Beyotime Biotechnology). After blocking for 1 h and 30 min, the membranes were treated with primary antibodies to cleaved caspase-3, phospho-ERK1^Thr202/Tyr204^/ERK2^Thr185/Tyr187^, BIRC5, β-catenin, BMI1, CD133, OCT4, nestin, or NGFR overnight at 4 °C. The membranes were then washed with TBST (Solarbio) and incubated with horseradish peroxidase-conjugated secondary antibodies (Beyotime Biotechnology) for 1 h at 25 °C. The protein bands were detected using a chemiluminescence detection kit (Beyotime Biotechnology), and the optical densities of protein signals were calculated using ImageJ software (National Institutes of Health, Bethesda, MD, USA), with β-actin as the loading control.

### Proliferation assays

Cell proliferation inhibition was assayed using the Cell Counting Kit-8 (Dojindo Molecular Technologies, Rockville, MD, USA) on an iMark™ Microplate Absorbance Reader (Bio-Rad, Hercules, CA, USA). Cells (1 × 10^5^ cells/mL) were plated and treated for 72 h in triplicate for each experimental condition. Each well was incubated with 10 μL of CCK8 reagent in 100 μL of cell culture medium, then the 96-well plate was incubated for 1 h at 37 °C in a humidified atmosphere containing 5% CO_2_. The absorbances were then measured at 450 nm, which were corrected relative to blank wells containing CCK8 reagent and medium only.

### Co-IP assays

The cells were cultured to 80%–90% confluence, and treated with drug regimens consisting of vemurafenib, dabrafenib, vemurafenib + trametinib, dabrafenib + trametinib, or DMSO. After a 72 h incubation with the small molecular weight drugs, the cell lysates were diluted with lysis buffer (NP-40, supplemented with protease inhibitors and phosphatase inhibitors), and incubated with 10 μg polyclonal rabbit anti-LGR5 (Sino Biological, Beijing, China) or 10 μg monoclonal mouse anti-HDGF (Proteintech), for 10–14 h at 4 °C with end-over-end mixing. Protein A/G-agarose beads (Beyotime Biotechnology) were then added and the reaction mixture was further mixed for 2 h at 4 °C. The immunoprecipitates were separated from the supernatants by centrifugation and washed with PBS containing 1% NP-40. The proteins were extracted from the agarose beads by boiling in 1× SDS gel loading buffer and resolved using 10% SDS-PAGE.

### Co-inhibition efficiency tests of *siHDGF* and *siLGR5*

A total of 800 μL cells were initially plated in 12-well plates (Corning, Corning, NY, USA) at a density of 2 × 10^5^ cells/mL for each treatment with the RPMI 1640 + 10% FBS culture medium. Then, 100 pmol siRNA of HDGF and LGR5, or a scrambled negative control siRNA, were co-transfected with Lipofectamine 3000™ into A375, H1299, or MHCC97H cells when the cell confluence reached 70%–80%. The total RNA of samples was extracted on the 3rd day after the siRNA transfection. The total RNA was reverse-transcribed into cDNA using the PrimeScript™ RT Master Mix (Takara, Shiga, Japan). Then, approximately 1 μg cDNA of each sample was added to the 2× Taq PCR Master Mix (Syngentech, Beijing, China) with corresponding primers to amplify HDGF, LGR5, and β-actin sequences. HDGF, LGR5, and β-actin primer sequences were obtained from PrimerBank (https://pga.mgh.harvard.edu/primerbank), where the PrimerBank IDs of HDGF, LGR5, and β-actin were 186928818c2, 24475886c1, and 4501885a1, respectively.

### Spheroid formation assays

A total of 100 μL of cells were initially plated in an ultra-low attachment 96-well plate (Corning) at a density of 2 × 10^5^ cells/mL in F12K/DMEM basic culture medium with 20 μg/mL EGF, 20 μg/mL bFGF, 5 μg/mL LIF, 10 μg/mL insulin, 1% N2, and 1% B27. After 24 h, the siRNAs of HDGF and LGR5 were transfected into A375, H1299, and MHCC97H cells according to the treatment groups, and after another 24 h, 2 μM vemurafenib and 2 μM vemurafenib + 10 nM trametinib were added to the A375 cells, and 5 μM dabrafenib and 5 μM dabrafenib + 100 nM trametinib were added to the H1299 and MHCC97H cells Then, spheroid images were captured by using a 4× microscope objective, every 2 days until the 7th day. The spheroid formation efficiency was evaluated by AnaSP and ReViSP, which are MATLAB programs that can calculate multiple morphological parameters of spheroids^47^, with the reconstruction of three-dimensional (3D) images, which reflected the stereoscopic morphology of the spheroids^48,49^.

### Immunofluorescence

A total of 4 × 10^4^ cells were initially plated in cover glasses that had been set in the bottom of a 24-well plate, and after 24 h, the siRNAs of HDGF and LGR5 were transfected into cells according to the treatment groups. After another 48 h, 2 μM vemurafenib or DMSO was added to the cells. After 72 h of drug treatment, the cells were fixed in 4% formaldehyde (Beyotime Biotechnology) in 1× TBS for 10 min at room temperature, permeabilized in 0.3% TritonX-100-TBS or Saponin-TBS for 10 min, washed in 0.1% Tween20-TBS (TBST) and incubated in blocking solution (Beyotime Biotechnology) for 1 h. The cells were then treated with primary antibodies overnight in TBST with 10% FBS at 4 °C, washed in TBST, and treated with secondary antibodies at 37 °C for 1 h in TBST. After washing, the cells were examined using an A1 confocal microscope (Nikon, Tokyo, Japan). Primary antibodies were anti-mouse-SOX2 (Sino Biological), anti-rabbit-KLF4 (Beyotime Biotechnology), anti-rabbit-Phospho-FAK (Beyotime Biotechnology), and anti-nestin (Beyotime Biotechnology).

### Ethical approval

Written informed consent was obtained from all patients, and the study protocol was approved by the Ethics Committee of the National Cancer Center/Center Hospital, Chinese Academy of Medical Sciences and Peking Union Medical College (Approval No. NCC20190-008).

## Results

### Construction of the pair-wise sgRNA library and the strategy for screening experiments

We selected 84 genes based on previous CRISPR/Cas9 single gene knockout and transcriptional activation screening studies^13,21^, and other mechanistic reports concerning the single key genes involved in MAPKi resistance (**Supplementary Table S3**)^7,11,12,14–16,18,19,24,27^. These genes function in different biological processes, including basic transcription regulation, mitotic progression, cell death regulation, regulation of GTPase activity, chromatin covalent modification, and the transmembrane receptor protein tyrosine kinase signaling pathway (**Supplementary Figure S2A**). We then used CRISPR-ERA to design sgRNAs that targeted the genes of interest, as well as a set of negative control sgRNAs (**Supplementary Table S4**)^50^.

**Figure 1:**
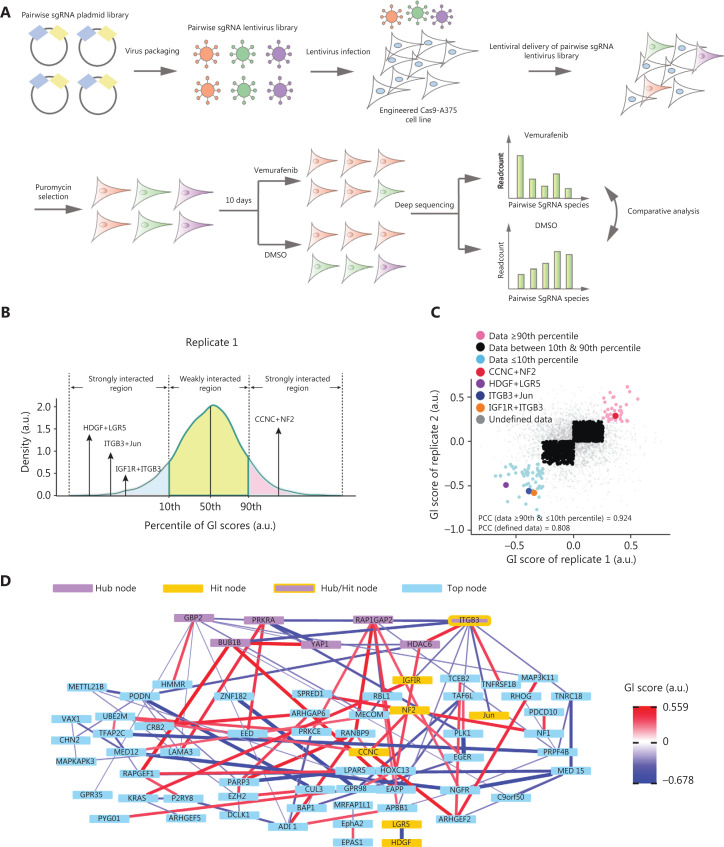
Screening combinatorial gene hits by using a pair-wise sgRNA library. (A) Functional screening using the pair-wise sgRNA library of the Cas9-A375 cell line. Cas9-A375 cells were infected with the pooled pair-wise sgRNA library and treated with vemurafenib or dimethyl sulfoxide. The abundances of pair-wise sgRNA combinations after different treatments were analyzed by paired-end deep sequencing. (B) The histogram distribution of averaged *GI* scores derived from replicate 1. *GI* scores below the 10th percentile or above the 90th percentile were recognized as strong interactions. (C) Correlation of *GI* scores of 2 independent replicates. Blue and pink dots represent the top and bottom 10% of reproducible *GI* scores; black dots indicate the middle 80% of reproducible *GI* scores; and gray dots denote undefined *GI* scores in 2 independent replicates. Four selected gene pairs are shown in red, green, blue, and orange. (D) The hierarchical network representation of the top listed *GI* score pairs. The thickness of edges indicates the GI strength. Blue and red designate the sensitizing and protective phenotypes, respectively. Purple nodes inside the yellow rectangle denote hub gene nodes. Blue nodes represent the top gene nodes. Yellow nodes were selected for experimental validation, as shown in subsequent figures.

To build the pair-wise sgRNA library, the synthesized and annealed sgRNA oligos were grouped into a negative control sgRNA pool and 3 positive sgRNA pools. The 3 positive sgRNA pools contained 3 sets of sgRNAs targeting 84 single genes (**Supplementary Figure S2B**). Each sgRNA pool was separately cloned into intermediate vectors with either the human U6 or 7SK promoter using the Golden Gate cloning method, to generate single sgRNA libraries with ~100× transformation coverage (**Supplementary Figure S2B**). To link the single-wise sgRNA libraries into pair-wise sgRNA libraries, 2 single sgRNA libraries were simultaneously cloned into the lentiviral vector by using a second Golden Gate reaction, to generate sublibraries with 10× transformation coverage (**Supplementary Figure S2B**). All sublibraries were mixed in equimolar amounts to generate the total pair-wise sgRNA library that contained more than 40,000 pair-wise sgRNA combinations targeting zero (negative control), 1 (single-wise), or 2 genes (pair-wise) among the 84 selected genes (**Supplementary Figure S2B**). Cas9-expressing A375 cells (Cas9-A375) were infected with a low titer of the pair-wise sgRNA library to increase the likelihood of single copy lentiviral integration into each genome (**Figure 1A**). After puromycin selection to ensure sufficient lentiviral integration and Cas9 knockout, infected A375 cells were treated with vemurafenib or DMSO for 10 days (**Figure 1A**). To assess the reproducibility of our high-throughput method, we independently performed the screening experiment twice. Then, the abundances of pair-wise sgRNA combinations in different cell populations were quantified by paired-end deep sequencing of 2 variable sgRNA spacer sequences. The abundances were used as a proxy for cell fitness under different treatments (**Figure 1A**). In addition, over 92% of sgRNA combinations were recovered in samples of the plasmid library, and cells from replicates 1 or 2 in the baseline day (**Supplementary Figure S2C**). We showed that the abundances of individual pair-wise sgRNA combinations in plasmid samples were highly correlated with those in either T0 sample, with Pearson’s correlation coefficient > 0.75 (**Supplementary Figure S2D and S2E**), and the plasmid frequencies of the 3 samples were also evenly distributed (**Supplementary Figure S1**).

### Phenotypic evaluation of the pair-wise sgRNA library

To quantify the growth phenotype of Cas9-A375 cells with different pair-wise sgRNA perturbations in response to vemurafenib treatment, we defined the ρ score, which was adapted from previous studies (see details in the Materials and Methods)^34^. When mutant Cas9-A375 cells showed resistance to vemurafenib treatment compared to wild-type cells, the ρ score was greater than 0. In contrast, a ρ score below 0 indicated that cells were more sensitive to vemurafenib treatment than wild-type cells. From the testing experiments above, we found that sgRNA driven by either U6 or 7SK could equally cause mutations in a given target gene (**Supplementary Figure S3**). We used the averaged ρ scores of pair-wise sgRNA combinations targeting the same gene or gene pair after excluding the minimal and maximal scores as the ρ score of the corresponding single gene or gene pair perturbation for further analyses. To identify strong growth phenotypes, we analyzed the distribution of obtained ρ scores and arbitrarily set the 10th and 90th percentiles as thresholds, selecting 68 gene pairs with strong sensitizing phenotypes and 159 gene pairs with strong protective phenotypes, respectively, for further analysis (**Supplementary Figure S4A and Supplementary Table S5**). In addition, we found that ρ scores below the 10th percentile threshold and above the 90th percentile threshold were well correlated in 2 screening replicates, with a Pearson’s correlation coefficient of 0.949 (**Supplementary Figure S4B**). We then constructed a network of identified gene pairs with strong ρ scores. We found 8 genes as the top 10% strong hubs, including 6 genes involved in the protective phenotype and 2 genes involved in the sensitizing phenotype (**Supplementary Figure S4C**).

### Calculation of GI scores from phenotypic scores

To effectively analyze the genetic interaction strength, we performed a linear regression analysis by using the ρ scores of 84 single gene perturbations versus the ρ scores of gene pair perturbations that shared a common bait gene (**Supplementary Figure S5A**). This strategy for calculating *GI* scores was based on the assumption that strong GIs are usually rare in large gene networks^38^. The linear fit represented the expected phenotype (expected) of gene pair perturbation, and *GI* scores of gene pair perturbations were calculated as the deviation from the expected phenotype to the observed phenotype (observed) (**Supplementary Figure S5A**). We observed a large number of gene pair perturbations resulting in small *GI* scores outside the linear fit with a 99% confidence interval, suggesting that further selection criteria may be needed to choose strong GI gene pairs (**Supplementary Figure S5A**).

To select strong and reliable GIs, we also analyzed the overall distribution of obtained *GI* scores. Similarly, we defined the 10th and 90th percentiles of the *GI* score distributions as arbitrary thresholds for selecting strong GIs (**Supplementary Figure S5B**). It has been reported that weak genetic interactions often produce a low correlation between *GI* scores derived from different screening replicates^45^. Thus, we analyzed the association of *GI* scores between 2 independent screening replicates. We found that 59 gene pairs below the 10th percentile threshold and 43 gene pairs above the 90th percentile threshold were highly consistent between the 2 replicates, with an overall Pearson’s correlation coefficient of 0.924 (**Supplementary Figure S5C and Supplementary Table S6**). In contrast, weak GIs were less correlated between 2 replicates compared to the strong GIs. To assay the relationships among these identified genes and thus identify important biomarkers^51^, we constructed a GI network by using the strong GI gene pairs (**Figure 1D**). Seven genes, including *ITGB3, GBP2, BUB1B, PRKRA, YAP1, RAP1GAP2*, and *HDAC6*, were identified as hubs with top ranked dense connectivities with other gene nodes (**Figure 1D**). Notably, only *RAP1GAP2* was recognized as the hub node in both networks constructed with strong *GI* scores and strong ρ scores (**Figure 1D and Supplementary Figure S4C**).

### Phenotypic confirmation of candidate genetic interaction pairs

**Figure 2:**
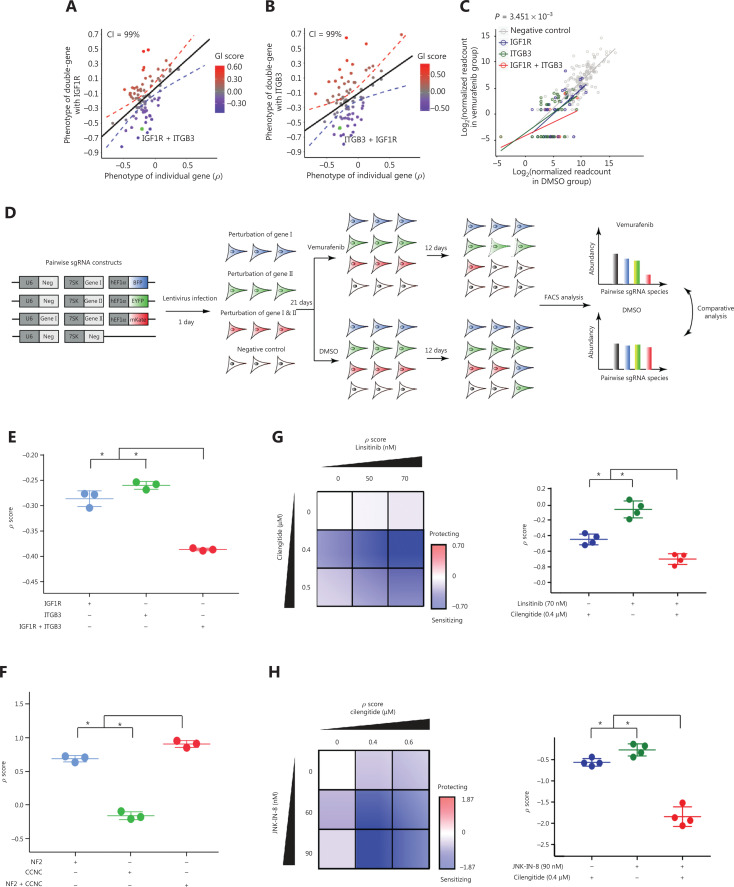
Combinatorial growth competition and small molecule intervention assays. (A, B) Scatter plots of ρ scores of pair-wise sgRNA perturbations against a common bait gene and single sgRNA perturbations. *GI* scores were calculated as the deviation from the observed ρ score to a linearly fitted value (black line). The dotted line denotes the 99% confidence interval. The color bar represents *GI* scores from negative (blue) to positive (red) effects. (C) Scatter plots of read count abundances of single and pair-wise sgRNA species in vemurafenib- and dimethyl sulfoxide-treated samples. Red dots, pair-wise sgRNA species; green and blue, single sgRNA species; and gray dots, negative control sgRNA. Linear fit lines for pair-wise (red), single (green, blue), and negative control (gray) sgRNA species are shown. Data were tested for significance using the 2-tailed Mann-Whitney U test. The *P*-value indicates the results of Mann-Whitney U tests comparing the distributions of ρ scores of the gene pair perturbation to those of the negative control. (D) Schematic illustration of the competitive growth assay. (E and F) Results of the combinatorial growth competition assay. The ρ scores were calculated by using the relative abundances of individual Cas9-A375 derivatives (see details in the Materials and Methods). The data are the mean ± s.d. (*N* = 3) from 3 independent replicates. “*” designates significant difference (*P* < 0.01) between single and pair-wise sgRNA perturbations tested by unpaired 2-tailed Student’s *t*-tests. (G, H) Cilengitide, linsitinib, and JNK-IN-8 are small molecular weight inhibitors of ITGB3, IGF1R, and JNK, respectively. A375 cells were treated with the indicated amounts of inhibitors. Vemurafenib resistance strength was calculated based on the number of live cells from different treatment groups. Data points are the mean ± s.d. (*N* = 4) from 3 independent replicates. “*” designates significant difference (*P* < 0.01) between single drug and combinatorial drug treatments tested by unpaired 2-tailed Student’s *t*-tests.

We confirmed that the *GI* scores of all 4 selected gene pairs (*ITGB3 + IGF1R, ITGB3 + Jun, NF2 + CCNC*, and *HDGF + LGR5*) fell outside the linear fit with a 99% confidence interval (**Figure 2A–2C, Supplementary Figure S6A and S6B, S6D and S6E, and S6G and S6H**). Subsequently, we compared the normalized read counts of pair-wise sgRNA perturbations to the normalized read counts of single sgRNA perturbations and negative control sgRNAs (**Figure 2C and Supplementary Figure S6C, S6F, and S6I**). We confirmed that pair-wise genetic perturbations of *ITGB3 + IGF1R* and *ITGB3 + Jun* resulted in strong sensitizing phenotypes, and perturbation of *NF2 + CCNC* caused a strong protective phenotype (**Figure 2C and Supplementary Figure S6C, S6F, and S6I**).

For gene pairs of *ITGB3 + IGF1R* and *NF2 + CCNC*, we engineered 3 Cas9-A375 cell lines that expressed either the EBFP or EYFP fluorescent protein along with a single sgRNA targeting individual genes or expressed 2 sgRNAs and the mKate fluorescent protein. The 3 engineered Cas9-A375 cell lines with either single or pair-wise gene perturbation(s) were mixed with Cas9-A375 cells in 4 equal proportions and cultured with vemurafenib or DMSO for up to 12 days (**Figure 2D**). By using flow cytometry analysis, we confirmed that perturbations of *ITGB3 + IGF1R* gene pairs generated a sensitizing growth phenotype (**Figure 2E and Supplementary Figure S7A**) and that perturbation of the *NF2 + CCNC* gene pair caused a protective growth phenotype (**Figure 2F and Supplementary Figure S7B**). Next, we assayed the growth phenotypes of A375 cells after treatment with clinically approved small molecular weight inhibitors, including cilengitide for ITGB3, linsitinib for IGF1R, and JNK-IN-8 for the downstream effector of Jun-c-Jun N-terminal kinase 1 (JNK). Consistent with our previous findings, when A375 cells were treated with a combinatorial treatment of varying amounts of cilengitide and linsitinib, a significant sensitizing growth phenotype and a strong synergistic effect were observed (**Figure 2G**). Additionally, because Jun lacks an inhibitor at the protein level, the small molecule JNK-IN-8 was used to inhibit JNK, which is the upstream regulator of Jun^52^. We confirmed that co-targeting JNK and ITGB3 also cooperatively sensitized A375 cells to vemurafenib (**Figure 2H**). We subsequently asked whether these combinatorial hits were also potentially applicable in other cancer types and whether the use of vemurafenib + trametinib or dabrafenib + trametinib plus the combinatorial adjuvant hits would achieve a superior effect compared with the sole use of vemurafenib or dabrafenib. We thus tested cell viabilities by using the combinatorial drug regimens of cilengitide + linsitinib and cilengitide + JNK-IN-8 across multiple cancer cell lines, including melanoma (MeWo), head and neck squamous carcinoma (FaDu), and breast cancer (MCF7) cells. The cell viability results showed that the melanoma MeWo cell line was sensitized by all 3 combinations, except cilengitide + JNK-IN-8 paired with vemurafenib + trametinib (**Supplementary Figure S8**). In contrast, FaDu was sensitized only by cilengitide + JNK-IN-8 paired with dabrafenib alone or dabrafenib + trametinib. However, in the breast cancer cell line MCF7, only cilengitide + JNK-IN-8 paired with dabrafenib produced sensitization (**Supplementary Figure S8**). Notably, we found that the combinatorial use of MAPKi had a minor function in enhancing the cooperative adjuvant combinatorial sensitizing effects. These results indicated that our pair-wise sgRNA screening strategy could be used to efficiently identify gene pairs with both sensitizing and protective phenotypes.

### The synergistic sensitizing mechanisms of combinatorial drug treatments

To understand the mechanisms responsible for the synergistic sensitization produced by using the drug combinations of vemurafenib + cilengitide + linsitinib and vemurafenib + cilengitide + JNK-IN-8, A375 cells were treated with 8 subcombinations of each group of triple drugs. Using qRT-PCR, we found that the relative mRNA expression of *E-cadherin* was dramatically elevated in cells treated with vemurafenib + cilengitide + linsitinib compared to cells treated with vemurafenib alone or cilengitide + linsitinib (**Supplementary Figure S9B**). In contrast, the mRNA expression of vimentin in A375 cells treated with vemurafenib + cilengitide + linsitinib sharply declined compared to that in A375 cells treated with cilengitide + linsitinib (**Supplementary Figure S9C**). These results indicated that a potential cellular transformation from the mesenchymal status to the epithelial status was induced in A375 cells with the administration of triple drugs (vemurafenib + cilengitide + linsitinib).

**Figure 3:**
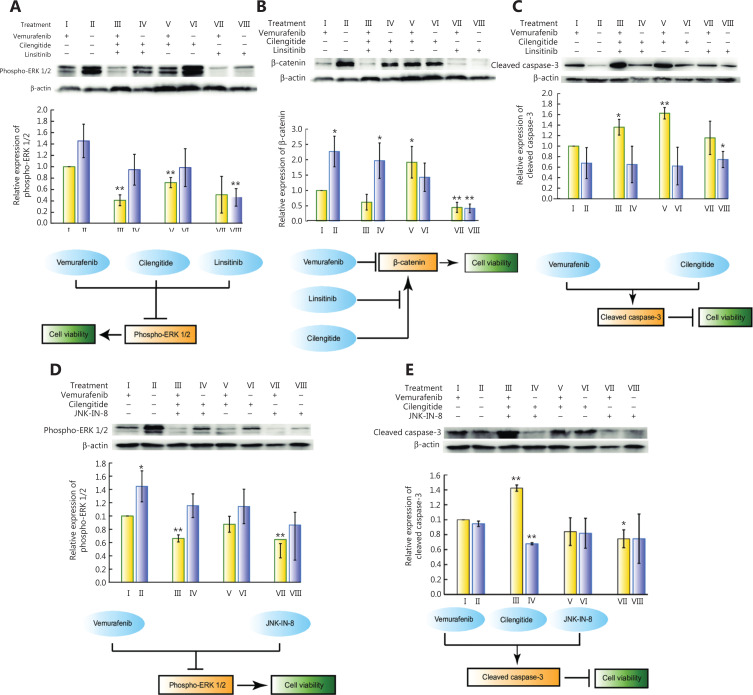
Cooperative sensitizing mechanisms of cilengitide + linsitinib and cilengitide + JNK-IN-8 during vemurafenib treatment. (A−C) The relative expressions of β-catenin, cleaved caspase-3, and phospho-ERK1/2 during different combination treatments with cilengitide + linsitinib with or without vemurafenib and its underlying cooperative mechanism. (D, E) The relative expressions of cleaved caspase-3 and phospho-ERK1/2 during different combination treatments with cilengitide + JNK-IN-8 with or without vemurafenib and its underlying cooperative mechanism. Bar plots are the mean ± s.d. from 3 independent replicates. “*” and “**” designate significant differences (*P* < 0.05 and 0.01, respectively) between the single drug vemurafenib treatment *vs.* combinatorial drug treatments tested by the unpaired 2-tailed Student’s *t*-test.

As a hub node in the genetic interaction network of vemurafenib resistance (**Figure 1D**), both ITGB3 and IGF1R are responsible for the reactivation of ERK1/2^23,53^. By using Western blot, we found that linsitinib was essential for repressing the expression of phospho-ERK1/2 (**Figure 3A**), suggesting that IGF1R rather than ITGB3 might be the main relay for the reactivation of ERK. In addition, vemurafenib blocked the activity of BRAF^V600E^. Notably, vemurafenib and linsitinib cooperatively inhibited the promotion of β-catenin by cilengitide (**Figure 3B**). Furthermore, cilengitide and vemurafenib synergistically enhanced the level of cleaved caspase-3 (**Figure 3C**). These results suggested that linsitinib + cilengitide cooperated to decrease the downstream oncogenic signal of BRAF^V600E^, cilengitide played a key role in inducing apoptosis with vemurafenib, and linsitinib limited the oncogenic effect of cilengitide in upregulating the expression of β-catenin.

We also examined the protein levels of cleaved caspase-3 and phospho-ERK1/2 in the 8 subcombinations of vemurafenib + cilengitide + JNK-IN-8, showing that among the groupings of these 3 drugs, vemurafenib + JNK-IN-8 epistatically reduced the expression of phospho-ERK1/2 relative to treatment with vemurafenib alone (**Figure 3D**). Triple drug treatment (vemurafenib + cilengitide + JNK-IN-8) had a considerable impact on the induction of cleaved caspase-3 (**Figure 3E**). These results showed that the cooperative sensitizing phenotype of cilengitide + JNK-IN-8 during vemurafenib pressure was achieved by the joint stimulation of apoptotic facilitators through co-targeting of ITGB3 + JNK and the inhibition of the oncogenic signal of phosphorylated ERK1/2 through targeting of JNK.

### Clinical relevance of combinatorial targets

**Figure 4:**
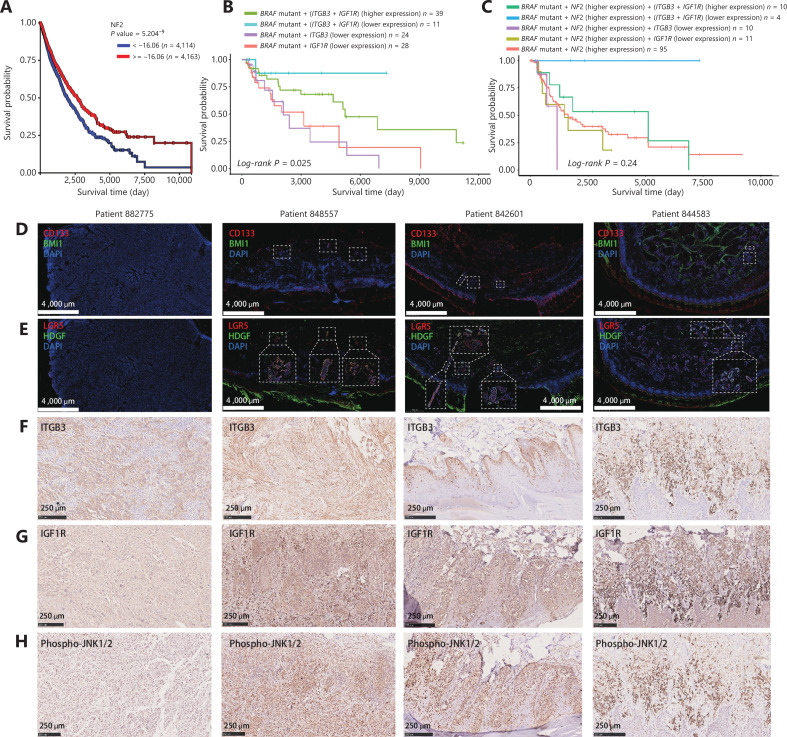
The clinical relevance of pair-wise sgRNA library-selected candidates. (A) Kaplan-Meier survival plot of melanoma patients according to the RNA expression level of *NF2* in the ICGC database. (B) Kaplan-Meier survival plot of BRAF-mutated melanoma patients according to the combination of *ITGB3* and *IGF1R* RNA expression levels from The Cancer Genome Atlas (TCGA) database. The statistical significance of differences in survival was evaluated by the log-rank test. (C) Kaplan-Meier survival plot of BRAF-mutated melanoma patients according to the combination of *NF2, ITGB3*, and *IGF1R* RNA expression levels from TCGA database. The statistical significance of differences in survivals was evaluated by the log-rank test. (D) Representative immunofluorescence (IF) micrographs showing CD133 (red) and BMI1 (green) expressions in patient-derived CD133^LOW^/BMI1^LOW^ or CD133^HIGH^/BMI1^HIGH^ melanoma samples 882775, 848557, 842601, and 844583, respectively. Scale bars, 1,000 μm (white). (E) Representative IF micrographs showing LGR5 (red) and HDGF (green) expressions in patient-derived CD133^LOW^ or CD133^HIGH^ melanoma samples 882775, 848557, 842601, and 844583. Scale bars, 1,000 μm (white). (F) Representative immunohistochemistry (IHC) micrographs showing ITGB3 expression in patient-derived CD133^LOW^/BMI1^LOW^ or CD133^HIGH^/BMI1^HIGH^ melanoma samples 882775, 848557, 842601, and 844583. Scale bars, 250 μm (black). (G) Representative IHC micrographs showing IGF1R expressions in patient-derived CD133^LOW^/BMI1^LOW^ or CD133^HIGH^/BMI1^HIGH^ melanoma samples 882775, 848557, 842601, and 844583. Scale bars, 250 μm (black). (H) Representative IHC micrographs showing phospho-JNK1/2 expression in patient-derived CD133^LOW^/BMI1^LOW^ or CD133^HIGH^/BMI1^HIGH^ melanoma samples 882775, 848557, 842601, and 844583. Scale bars, 250 μm (black).

To bridge the gap between our *in vitro* results and the demands of clinical applications, we aimed to examine the expression of genes and proteins from the candidate synergistic pairs in melanoma patients, which might have a potential impact on current clinical practices for melanoma treatment. Because NF2 was reported as a vital tumor suppressor^54^, we constructed a Kaplan-Meier plot of data from 8,277 patients in the ICGC^55^ melanoma database to identify the relationship of *NF2* gene expressions in melanomas and patient survivals (**Figure 4A**). We found that a decreased Neurofibromin 2 (*NF2*) mRNA level was associated with a shortened survival in melanoma patients (P_NF2_ < 5.204 × 10^-9^). It has been reported that integrin subunit beta 3 (ITGB3) is positively related to a higher frequency of malignant melanomas than benign lesions, and insulin-like growth factor 1 receptor was found to be involved in adaptive bypass of BRAF^V600E^ inhibition^44,56^. After analyses of the clinical survival information of melanoma patients with *BRAF* mutations using TCGA database^57^, we performed survival analyses that associated the discretized *NF2, ITGB3*, and *IGF1R* expression status (higher, high, medium, low, and lower) of every individual patient with overall survivals. Notably, we found that *BRAF* mutation patients with lower expressions of *ITGB3* and *IGF1R* exhibited a significantly higher survival than patients in the other groups (**Figure 4B and 4C**).

Using CD133 and BMI1 as biological markers of cancer malignancy^58,59^, we found that melanoma patient tissues with high grade expression of CD133 and BMI1 were associated with high levels of ITGB3, IGF1R, and JNK, as well as co-expression of LGR5 + HDGF (**Figure 4D–4H**), where these hits were selected from the pair-wise sgRNA screening library and calculated as efficient synergistic pairs for sensitizing BRAFi resistance. We also found that G-protein coupled receptor 5 (LGR5) and hepatoma-derived growth factor (HDGF) were usually co-expressed with CD133. Because LGR5 is a biomarker for hair follicle stem cells, it plays a role in sustaining skin homeostasis, and the cells expressing this protein are thought to be a subset of cells with high stemness that contribute to tumor development^60,61^. We also found that HDGF and LGR5 co-localized morphologically in the microvasculature regions, suggesting that HDGF and LGR5 might both be involved in tumor angiogenesis and cancer stemness.

Thus, we were particularly interested in the combinatorial targets of ITGB3 + IGF1R, ITGB3 + JNK, and HDGF + LGR5, not only because of their high clinical significance, but also because ITGB3 + IGF1R and ITGB3 + JNK are FDA-approved drug targets, and HDGF + LGR5 potentially interact in a physical complex to contribute to tumor angiogenesis and cancer stemness.

### HDGF and LGR5 specifically respond to MAPK inhibitors as protein complexes, and complex formation is regulated by cilengitide + linsitinib and cilengitide + JNK-IN-8

**Figure 5:**
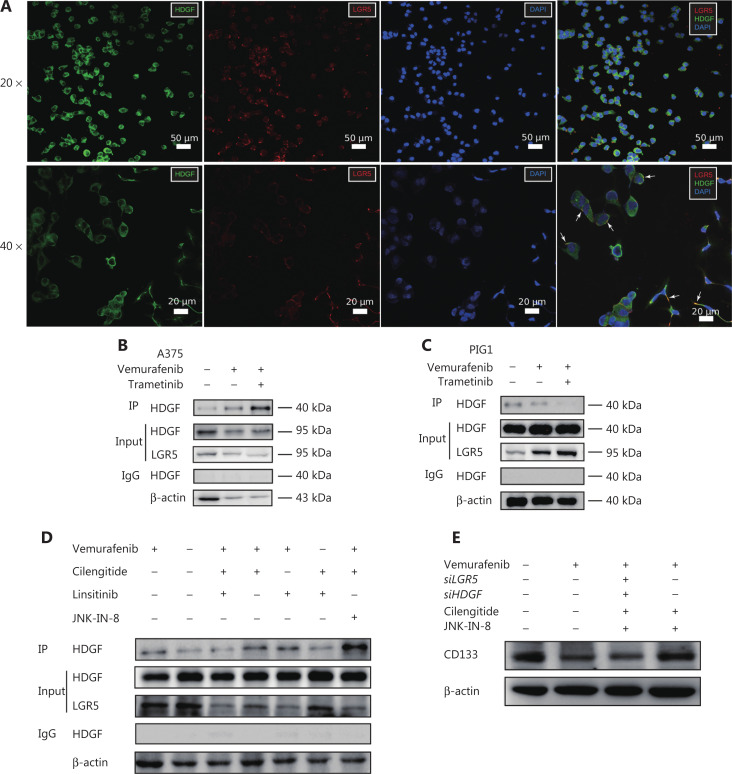
HDGF and LGR5 form a protein complex to respond to MAPKi stress, and the formation of the complex is regulated by cilengitide + linsitinib and cilengitide + JNK-IN-8. (A) Representative immunofluorescence (IF) micrographs showing HDGF (green) and LGR5 (red) expressions in A375 cells. Scale bars, 10 μm (white). (B) Immunoprecipitation (IP) assays with anti-LGR5 antibody extracts from A375 cells expressing endogenous HDGF exposed to dimethyl sulfoxide (DMSO), BRAFi, or BRAFi + MEKi for 72 h. (C) IP assays with anti-LGR5 antibody extracts from SK-MEL-28 cells expressing endogenous HDGF exposed to DMSO, BRAFi, or BRAFi + MEKi for 72 h. (D) IP assays with anti-LGR5 antibody extracts from A375 cells expressing endogenous HDGF exposed to different drug combinations for 72 h. (E) Western blot showing CD133 levels in DMSO-, vemurafenib-, vemurafenib + JNK-IN-8 + cilengitide + *siLGR5* + *siHDGF*-, and vemurafenib + JNK-IN-8 + cilengitide-treated A375 cell samples.

HDGF has been implicated in cancer cell proliferation and angiogenesis^62,63^. However, it has been usually studied as a nuclear targeting mitogen that exerts its effect through internalization^64^. How HDGF regulates intracellular signals by interacting with cell surface receptors remains unknown. We found that HDGF and LGR5 usually co-localized in the cellular polarized tip apex of the A375 cell membrane, especially in morphologically mesenchymal-like cells (**Figure 5A**). We further showed that HDGF physically interacted with the cell surface protein, LGR5, in A375 cells with or without vemurafenib + trametinib treatment or vemurafenib treatment alone. However, the abundance of the HDGF-LGR5 complex increasingly responded to MAPKi stress in A375 cells (**Figure 5B**). Moreover, the A549 and H1299 cell lines also showed highly active complex formation of LGR5-HDGF during dabrafenib treatment (**Supplementary Figure S10**). These data suggested that HDGF may physically associate with LGR5 as a response mechanism to the stress of MAPK signaling inhibition. In contrast to these results, a normal melanocyte cell line, PIG1, showed decreased HDGF-LGR5 complex formation in response to the addition of vemurafenib + trametinib or vemurafenib alone (**Figure 5C**), and the cell viability of PIG1 stalled after 72 h with the adjuvant perturbation of HDGF and LGR5 (**Supplementary Figure S11**), suggesting that the complex might be essential for cell viability during MAPKi stress. However, we found that vemurafenib + cilengitide + linsitinib decreased HDGF-LGR5 complex formation, whereas complex formation was dramatically increased when vemurafenib + cilengitide was added along with the MAPK inhibitor, JNK-IN-8 (**Figure 5D**).

We also found that the protein level of CD133 was increased when A375 cells were treated with vemurafenib + cilengitide + JNK-IN-8, but the effective co-inhibition of HDGF and LGR5 in A375 cells abrogated the CD133 expression stimulated by vemurafenib + cilengitide + JNK-IN-8 (**Figure 5E, Supplementary Figure S12, and Supplementary Table S7**). These results suggested that the expression of CD133 may be associated with the formation of the LGR5-HDGF protein complex.

In summary, a potential relationship was found between responsive HDGF-LGR5 complex formation and emerging MAPKi resistance. These results indicated that LGR5 and HDGF might be critical adaptive mechanisms in the process of MAPKi resistance.

### Disruption of both HDGF and LGR5 synergistically represses the emergence of BRAFi-responsive oncogenic signals

**Figure 6:**
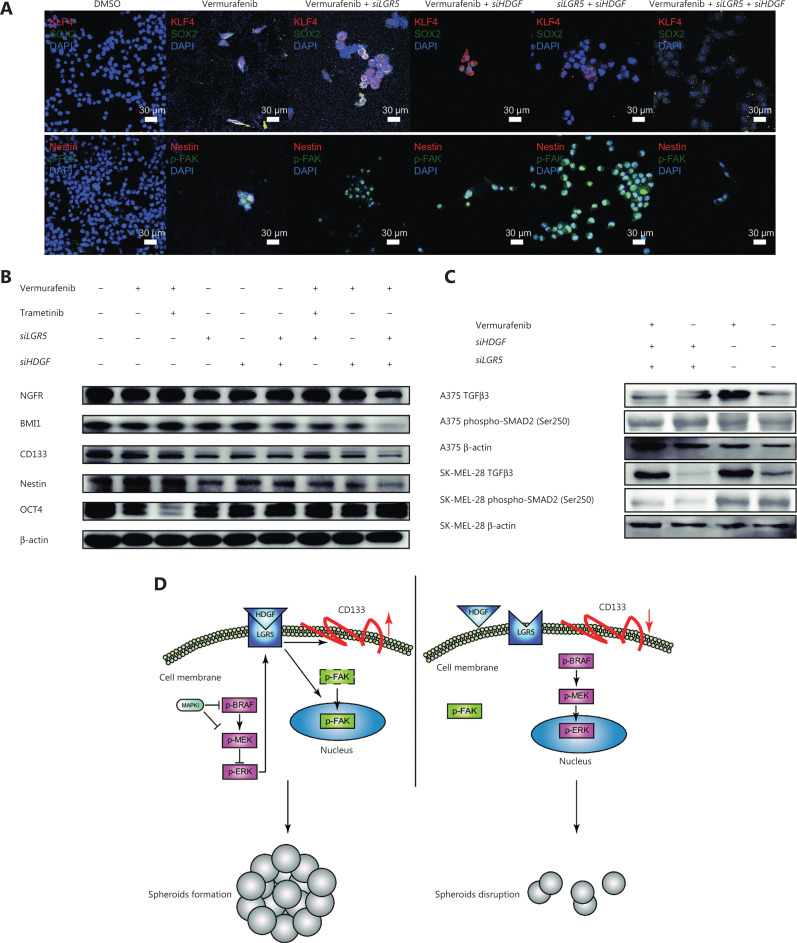
The disruption of both HDGF and LGR5 synergistically represses the emergence of BRAFi-responsive oncogenic signals. (A) Representative immunofluorescence micrographs showing KLF4 (red) and SOX2 (green) expressions, β-catenin (red), and CD133 (green) expressions, and nestin (red) and phospho-FAK (green) expressions in cells treated with dimethyl sulfoxide (DMSO), vemurafenib, vemurafenib + *siLGR5*, vemurafenib + *siHDGF*, *siLGR5* + *siHDGF*, or vemurafenib + *siLGR5* + *siHDGF*. Scale bars, 10 μm (white). (B) Western blot showing OCT4, NGFR, nestin, BMI1, and CD133 levels in DMSO-, vemurafenib-, vemurafenib + *siLGR5*-, vemurafenib + *siHDGF*-, *siLGR5* + *siHDGF*-, and vemurafenib + *siLGR5* + *siHDGF*-treated A375 cell samples. (C) Western blot showing TGFβ3 and phospho-SMAD2 (Ser250) levels in vemurafenib + *siHDGF* + *siLGR5*-, *siHDGF* + *siLGR5*-, vemurafenib-, and DMSO-treated A375 or SK-MEL-28 cell samples. (D) A proposed model for adaptive HDGF-LGR5 drug resistance to MAPK inhibitors.

Kruppel-like factor 4 (KLF4) and sex-determining region Y-box 2 (SOX2) have been reported as essential transcription factors that maintain CSC stemness and invasion activity in melanomas, in addition to many other cancer cells^65–71^. We further observed that digenic knockdown of *HDGF* and *LGR5* inhibited the nuclear localization of KLF4 (**Figure 6A**). In addition, SOX2 could also be essential for the preservation of cancer stemness (Rybak and Tang, 2013), and the heterodimerization of KLF4 and SOX2 is an important indicator of the initiation of CSC pluripotency^67^. We found that co-localization of KLF4-SOX2 was predominantly abrogated (**Figure 6A**). It was also equally important that phospho-FAK, whose nuclear localization has been shown to be an indicator of accelerated cell proliferation and enhanced cell survival^72^, was excluded from the cell nucleus in the tri-drug group (**Figure 6A**). We also observed that the expression of nestin, another CSC marker that indicates poor prognosis^73,74^, was decreased when *HDGF* and *LGR5* were simultaneously knocked down and vemurafenib was added (**Figure 6B**). Consistently, BMI1, CD133, and NGFR were downregulated by *HDGF* and *LGR5* double knockdown with exposure to BRAFi in A375 cells (**Figure 6B**). However, the combination of vemurafenib + trametinib was the only drug pair that downregulated the expression of OCT4, but not other CSC markers, implying its limited function in cancer stemness inhibition. Together, these results suggested that HDGF and LGR5 might play a necessary role in the responsive activation of stemness signals and induction of MAPKi resistance.

Typically, TGFβ3 is a positive modulator of the PI3-kinase/AKT signaling pathway and a bypass signaling molecule promoting survival when MAPK signaling is inhibited^75^. Moreover, phospho-SMAD2, a tumor suppressor in melanomas^76^, was found to be essential for nelfinavir to sensitize MAPKi resistance by repressing PAX3-mediated upregulation of MITF^77^. We found that phospho-SMAD2 was downregulated in SK-MEL-28 cells by the pair-wise perturbation of *HDGF* and *LGR5* (**Figure 6C**), and we did not observe complex formation between LGR5 and HDGF in SK-MEL-28 cells (**Supplementary Figure S13**). In contrast, phospho-SMAD2 was not affected by the pair-wise perturbation of *HDGF* and *LGR5* in A375 cells (**Figure 6C**). Together, these results suggested that HDGF-LGR5 complex formation might be a protective mechanism allowing specific kinds of tumor cells to sense MAPK repression, and thus strengthen tumor stemness for enhanced survival (**Figure 6D**).

### BRAFi with adjuvant genetic perturbation of *HDGF-LGR5* sensitizes spheroid formation in cancer cells

**Figure 7:**
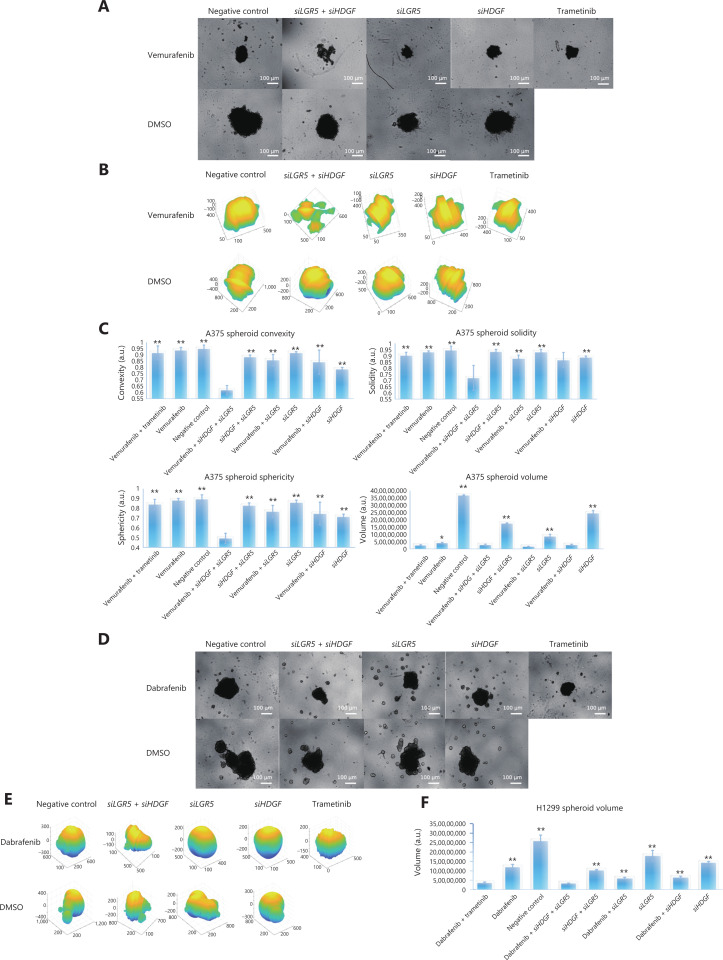
Pair-wise inhibition of *HDGF* and *LGR5* with MAPKi affected the efficiency of three-dimensional (3D) spheroid formation in cancer cells. (A) Images of A375 spheroids during drug perturbations in different combinations. Images were captured with a 4× microscope objective. (B) The 3D reconstruction images of A375 spheroids. (C) Estimated convexity, solidity, sphericity, and volume of A375 spheroids with combinatorial genetic perturbations of *HDGF* and *LGR5* with or without MAPKi. Bar plots show the mean ± s.d. (*N* = 3) from 3 independent replicates. “*” and “**” designate significant differences (*P* < 0.05 and 0.01, respectively) between the combinatorial drug regimen of vemurafenib + *siHDGF* + *siLGR5* and other drug treatments using the unpaired 2-tailed Student’s *t*-test. (D) Images of H1299 spheroids during drug perturbations in different combinations. Images were captured with a 4× microscope objective. (E) The 3D reconstruction images of H1299 spheroids. (F) Estimated volume of H1299 spheroids with combinatorial genetic perturbations of *HDGF* and *LGR5* with or without MAPKi. Bar plots show the mean ± s.d. (*N* = 3) from 3 independent replicates. “**” designates significant difference (*P* < 0.01) between the combinatorial drug regimens of vemurafenib + *siHDGF* + *siLGR5* and other drug treatments using the unpaired 2-tailed Student’s *t*-test.

Because LGR5 has been considered a specific stem cell marker within the hair follicle and intestine^60,78^, and to validate the MAPKi sensitizing effect of co-inhibition of *HDGF* and *LGR5*, we determined the spheroid formation ability in A375 cells and expanded our investigated cancer types to hepatoma and NSCLC cancer cells, which could better illustrate the stemness maintenance property of *LGR5*. We therefore used the AnaSP and ReViSP evaluation methods to analyze the spheroid parameters and then produced 3D image reconstructions^47,48^. The spheroid parameters were analyzed mainly in terms of volume, convexity, solidity, sphericity, and quantity. We found that spheroids originating from different cancers exhibited different morphological patterns. For example, A375 and H1299 grew as a single spheroid, but MHCC97H was composed of many individualized clones (**Figure 7A–7F and Supplementary Figure S14**). For A375 cells, we observed that the volumes of spheroids during treatment with vemurafenib + *siHDGF* + *siLGR5*, vemurafenib + *siHDGF*, vemurafenib + *siLGR5*, and vemurafenib + trametinib were not significantly different. However, the volumes associated with these combinations were decreased compared to those of other combinatorial groups, including the single use vemurafenib group (**Figure 7B and 7C**). We next analyzed the spheroid parameters of convexity, solidity, and sphericity to evaluate the morphological quality of spheroid formation^48^, and found that vemurafenib + *siHDGF* + *siLGR5* disrupted the A375 spheroid morphology by influencing convexity, solidity, and sphericity, when compared to other groups (**Figure 7B and 7C**). In H1299 cells, we found that dabrafenib + *siHDGF* + *siLGR5* and dabrafenib + trametinib resulted in equal decreases in spheroid volumes (**Figure 7D and 7E**). Simultaneously, these two combinations achieved the highest efficiency in controlling the volume of spheroids among these combinations of perturbations (**Figure 7E**). Notably, the use of 5 μM dabrafenib or 5 μM dabrafenib + 100 nM trametinib had an equivalent minor impact on the prohibition of MHCC97H spheroid counts and areas, when compared to the negative control group. However, the MHCC97H spheroids exhibited remarkable reductions in quantity and area in the *HDGF + LGR5* interference group when treated with dabrafenib (**Supplementary Figure S14**). Together, these results showed that pair-wise genetic perturbation of *HDGF* and *LGR5* with the addition of MAPKi efficiently disrupted the formation of tumor spheroids in the 3D growth environment.

## Discussion

Using conventional strategies of dissecting vemurafenib resistance in BRAF^V600E^ melanomas, various key players in multiple signaling pathways have been identified^10,14,24,31^. Because of the complexity of BRAF^V600E^ melanomas in response to vemurafenib intervention^79,80^, it is essential to use a systematic approach to analyze genetic interactions between important gene nodes. To accomplish this goal, we developed a CRISPR/Cas9-based pair-wise sgRNA functional screening and data mining platform, enabling us to systematically evaluate paired functional interactions among 84 genes involved in vemurafenib resistance. Although inhibiting a single target gene, such as *ROCK1* and *NGFR*, can sensitize vemurafenib-resistant melanoma cells^14,24^, our results showed that pair-wise perturbation of *ROCK1 + NF2* or *NGFR + NF2* conferred a strong protective phenotype during vemurafenib treatment (**Supplementary Figure S5A and S5B**). Furthermore, our identified drug combinations (cilengitide + linsitinib and cilengitide + JNK-IN-8) strongly sensitized A375 cells to vemurafenib single drug intervention. These combinations have not previously been reported as effective adjuvant regimens for overcoming drug resistance in tumors. In conclusion, these results highlighted the importance of studying genetic interactions among the genes involved in drug resistance.

While ρ scores quantified the growth phenotypes of sgRNA perturbations, *GI* scores reflected the extent of enhancement or inhibition relationships between 2 genes in response to drug treatment. Both types of information could be useful and are sometimes complementary to each other. Although the *GI* score alone may offer insights into strongly interacting genes, the ρ score, but not the *GI* score, can provide information on whether these strongly interacting genes are essential in the drug resistance phenotype. Consistent with previous findings^38^, we showed that weak genetic interactions displayed a low correlation among different screening replicates. In our study, the number of gene pairs with strong *GI* scores was smaller than the number of pairs with strong ρ scores, suggesting that genetic interactions may be more sensitive to experimental settings than cell growth phenotypes. Combining genetic interactions and growth phenotypes may therefore provide insightful information regarding cellular responses to drug interventions.

This study found that cilengitide + linsitinib and cilengitide + JNK-IN-8 sensitized A375 cells to vemurafenib. Although both drug combinations shared a common target, the drug cooperation mechanisms might differ, especially in their downstream effects on HDGF and LGR5 complex formation. Identifying multiple drug combinations for different genetic conditions is therefore necessary to provide effective cancer therapy *via* mutual neutralization of side effects caused by individual drug treatments^81^ and cooperative inhibition of multiple key targets to induce drug resistance. More importantly, because drug resistance has been frequently used in clinical practice during treatment with vemurafenib, dabrafenib, vemurafenib + trametinib, and dabrafenib + trametinib^82,83^, finding a therapeutic solution that overcomes the setbacks encountered with the current treatments at the molecular level may prolong patient survival. In the present study, we showed that the combined use of vemurafenib + trametinib blocked the expression of OCT4, which also plays a role in the maintenance of normal somatic stem cells^84^; however, CSC markers such as NGFR, BMI1, CD133 and nestin were not downregulated in the vemurafenib + trametinib group (**Figure 6B**), although these highly malignant factors have been thought to be the cause of tumorigenicity and cancer recurrence^14,74,81,85,86^. Thus, comparing the mechanistic molecular differences among different therapeutic combinations is advantageous for identifying tumor vulnerabilities when resistance arises. It is well-known that LGR5 is one of the best prognostic biomarkers for drug resistance or tumor relapse for many types of cancers^87–89^ and has been usually reported as an activator of the Wnt/β-catenin signaling pathway^90^.

However, the abundance of nuclear-localized HDGF is considered a remarkable prognostic factor in various cancers^91,92^, but its function in cancer has never been identified. In the present study, we proposed a novel molecular mechanism involving its prognostic significance, suggesting that LGR5 formed a protein complex with HDGF on the cell membrane, to function as a MAPKi response element, and promote cell survival by enhancing cancer stemness and the nuclear localization of FAK to protect tumor cells against MAPKi agents. This adaptive complex-forming mechanism is a newly discovered function that differs from the previously well characterized roles of LGR5 in the R-spondin-LGR5-Wnt/β-catenin axis and the previously defined functional mitogenic role of HDGF in the nucleus^91,92^. To the best of our knowledge, this is the first report of an adaptive protein complex formation mechanism that affects resistance to MAPKi in cancer cells, so it is important to characterize the protein interaction interfaces between HDGF and LGR5 in future studies to develop potential drugs to block the formation of the HDGF-LGR5 protein complex.

## Conclusions

In summary, we used a pooled pair-wise sgRNA screening and data analysis pipeline to comprehensively study the genetic interactions involved in vemurafenib resistance, which may facilitate an understanding of vemurafenib resistance mechanisms in many types of cancer cells. In addition, our strategy to identify sensitizing and protective gene pairs led to the discovery of novel combinatorial targets with clinical relevance, especially for the drug regimen of cilengitide + linsitinib and genotype combination of *HDGF-+ LGR5-*, which were found to be potent in overcoming MAPKi resistance in multiple cancer cells, which provided novel biological insights into a responsive protein complex formation mechanism that can adapt cancer cells to environmental stress. Combined with the clinical sample data, we propose that these insights can provide therapeutic opportunities.

## References

[1] Cantwell-Dorris ER, O’Leary JJ, Sheils OM (2011). BRAFV600E: implications for carcinogenesis and molecular therapy. Mol Cancer Ther.

[2] Das Thakur M, Salangsang F, Landman AS, Sellers WR, Pryer NK, Levesque MP (2013). Modelling vemurafenib resistance in melanoma reveals a strategy to forestall drug resistance. Nature.

[3] Joseph EW, Pratilas CA, Poulikakosb PI, Tadib M, Wangd W, Taylord BS (2010). The RAF inhibitor PLX4032 inhibits ERK signaling and tumor cell proliferation in a V600E BRAF selective manner. Proc Natl Acad Sci.

[4] Lu H, Liu S, Zhang G, Kwong LN, Zhu Y, Miller JP (2016). Oncogenic BRAF-mediated melanoma cell invasion. Cell Rep.

[5] Ravnan MC, Matalka MS (2012). Vemurafenib in patients with BRAF V600E mutation-positive advanced melanoma. Clin Ther.

[6] Alonso SR, Tracey L, Ortiz P, Perez-Gomez B, Palacios J, Pollan M (2007). A high-throughput study in melanoma identifies epithelial-mesenchymal transition as a major determinant of metastasis. Cancer Res.

[7] Carbone M, Yang H, Pass HI, Krausz T, Testa JR, Gaudino G (2013). BAP1 and cancer. Nat Rev Cancer.

[8] Easwaran H, Tsai HC, Baylin SB (2014). Cancer epigenetics: tumor heterogeneity, plasticity of stem-like states, and drug resistance. Mol Cell.

[9] Hann SR (2014). MYC Cofactors: molecular switches controlling diverse biological outcomes. Cold Spring Harb Perspect Med.

[10] Holohan C, Van Schaeybroeck S, Longley DB, Johnston PG (2013). Cancer drug resistance: an evolving paradigm. Nat Rev Cancer.

[11] Ji Z, Kumar R, Taylor M, Rajadurai A, Marzuka-Alcala A, Chen YE (2013). Vemurafenib synergizes with nutlin-3 to deplete survivin and suppresses melanoma viability and tumor growth. Clin Cancer Res.

[12] Kim MH, Kim J, Hong H, Lee S-H, Lee J-K, Jung E (2016). Actin remodeling confers BRAF inhibitor resistance to melanoma cells through YAP/TAZ activation. EMBO J.

[13] Konermann S, Brigham MD, Trevino AE, Joung J, Abudayyeh OO, Barcena C (2015). Genome-scale transcriptional activation by an engineered CRISPR-Cas9 complex. Nature.

[14] Lehraiki A, Abbe P, Cerezo M, Rouaud F, Allegra M, Kluza J (2015). Increased CD271 expression by the NF-kB pathway promotes melanoma cell survival and drives acquired resistance to BRAF inhibitor vemurafenib. Cell Discov.

[15] Miao B, Ji Z, Tan L, Taylor M, Zhang J, Choi HG (2014). EPHA2 is a mediator of vemurafenib resistance and a novel therapeutic target in melanoma. Cancer Discov.

[16] Nakanishi Y, Seno H, Fukuoka A, Ueo T, Yamaga Y, Maruno T (2012). Dclk1 distinguishes between tumor and normal stem cells in the intestine. Nat Genet.

[17] Nazarian R, Shi H, Wang Q, Kong X, Koya RC, Lee H (2010). Melanomas acquire resistance to B-RAF(V600E) inhibition by RTK or N-RAS upregulation. Nature.

[18] Peng U, Wang Z, Pei S, Ou Y, Hu P, Liu W (2017). ACY-1215 accelerates vemurafenib induced cell death of BRAF-mutant melanoma cells via induction of ER stress and inhibition of ERK activation. Oncol Rep.

[19] Roesch A, Vultur A, Bogeski I, Wang H, Zimmermann KM, Speicher D (2013). Overcoming intrinsic multidrug resistance in melanoma by blocking the mitochondrial respiratory chain of slow-cycling JARID1B high cells. Cancer Cell.

[20] Rossman KL, Der CJ, Sondek J (2005). GEF means go: turning on Rho GTPases with guanine nucleotide-exchange factors. Nat Rev Mol Cell Biol.

[21] Shalem O, Sanjana NE, Hartenian E, Shi X, Scott DA, Mikkelsen TS (2014). Genome-scale CRISPR-Cas9 knockout screening in human cells. Science.

[22] Shi H, Moriceau G, Kong X, Lee M-K, Lee H, Koya RC (2012). Melanoma whole-exome sequencing identifies V600E B-RAF amplification-mediated acquired B-RAF inhibitor resistance. Nat Commun.

[23] Smalley KSM (2003). A pivotal role for ERK in the oncogenic behaviour of malignant melanoma?. Int J Cancer.

[24] Smit MA, Maddalo G, Greig K, Raaijmakers LM, Possik PA, van Breukelen B (2014). ROCK1 is a potential combinatorial drug target for BRAF mutant melanoma. Mol Syst Biol.

[25] Villicaña C, Cruz G, Zurita M (2014). The basal transcription machinery as a target for cancer therapy. Cancer Cell Int.

[26] Voulgari A, Pintzas A (2009). Epithelial-mesenchymal transition in cancer metastasis: mechanisms, markers and strategies to overcome drug resistance in the clinic. Biochim Biophys Acta.

[27] Zingg D, Julien Debbache, Schaefer SM, Tuncer E, Frommel SC, Cheng P (2015). The epigenetic modifier EZH2 controls melanoma growth and metastasis through silencing of distinct tumour suppressors. Nat Commun.

[28] Liu X, Vorontchikhina M, Wang Y-L, Faiola F, Martinez E (2008). STAGA recruits mediator to the MYC oncoprotein to stimulate transcription and cell proliferation. Mol Cell Biol.

[29] Shim H, Chun YS, Lewis BC, Dang CV (1998). A unique glucose-dependent apoptotic pathway induced by c-Myc. Proc Natl Acad Sci.

[30] Yuneva M, Zamboni N, Oefner P, Sachidanandam R, Lazebnik Y (2007). Deficiency in glutamine but not glucose induces MYC-dependent apoptosis in human cells. J Cell Biol.

[31] Alcalá AM, Flaherty KT (2012). BRAF inhibitors for the treatment of metastatic melanoma: clinical trials and mechanisms of resistance. Clin Cancer Res.

[32] Rizos H, Menzies AM, Pupo GM, Carlino MS, Fung C, Hyman J (2014). BRAF inhibitor resistance mechanisms in metastatic melanoma: spectrum and clinical impact. Clin Cancer Res.

[33] Dixon SJ, Costanzo M, Baryshnikova A, Andrews B, Boone C (2009). Systematic mapping of genetic interaction networks. Annu Rev Genet.

[34] Bassik MC, Kampmann M, Lebbink RJ, Wang S, Hein MY, Poser I (2013). A systematic mammalian genetic interaction map reveals pathways underlying ricin susceptibility. Cell.

[35] Horn T, Sandmann T, Fischer B, Axelsson E, Huber W, Boutros M (2011). Mapping of signaling networks through synthetic genetic interaction analysis by RNAi. Nat Methods.

[36] Du D, Roguev A, Gordon DE, Chen M, Chen S-H, Shales M (2017). Genetic interaction mapping in mammalian cells using CRISPR interference. Nat Methods.

[37] Guo Y, Bao C, Ma D, Cao Y, Li Y, Xie Z (2019). Network-based combinatorial CRISPR-Cas9 screens identify synergistic modules in human cells. ACS Synth Biol.

[38] Han K, Jeng EE, Hess GT, Morgens DW, Li A, Bassik MC (2017). Synergistic drug combinations for cancer identified in a CRISPR screen for pairwise genetic interactions. Nat Biotechnol.

[39] Wong ASL, Choi GCG, Cui CH, Pregernig G, Milani P, Adam M (2016). Multiplexed barcoded CRISPR-Cas9 screening enabled by CombiGEM. Proc Natl Acad Sci.

[40] Xue Y, Martelotto L, Baslan T, Vides A, Solomon M, Mai TT (2017). An approach to suppress the evolution of resistance in BRAF V600E-mutant cancer. Nat Med.

[41] Welsh SJ, Rizos H, Scolyer RA, Long GV (2016). Resistance to combination BRAF and MEK inhibition in metastatic melanoma: where to next?. Eur J Cancer.

[42] Shibue T, Weinberg RA (2017). EMT, CSCs, and drug resistance: the mechanistic link and clinical implications. Nat Rev Clin Oncol.

[43] Irvine M, Stewart A, Pedersen B, Boyd S, Kefford R, Rizos H (2018). Oncogenic PI3K/AKT promotes the step-wise evolution of combination BRAF/MEK inhibitor resistance in melanoma. Oncogenesis.

[44] Villanueva J, Vultur A, Lee JT, Somasundaram R, Fukunaga-Kalabis M, Cipolla AK (2010). Acquired resistance to BRAF inhibitors mediated by a RAF kinase switch in melanoma can be overcome by cotargeting MEK and IGF-1R/PI3K. Cancer Cell.

[45] Kampmann M, Bassik MC, Weissman JS (2013). Integrated platform for genome-wide screening and construction of high-density genetic interaction maps in mammalian cells. Proc Natl Acad Sci.

[46] Ma D, Peng S, Xie Z (2016). Integration and exchange of split dCas9 domains for transcriptional controls in mammalian cells. Nat Commun.

[47] Piccinini F (2015). AnaSP: a software suite for automatic image analysis of multicellular spheroids. Comput Methods Programs Biomed.

[48] Zanoni M, Piccinini F, Arienti C, Zamagni A, Santi S, Polico R (2016). 3D tumor spheroid models for in vitro therapeutic screening: a systematic approach to enhance the biological relevance of data obtained. Sci Rep.

[49] Piccinini F, Tesei A, Arienti C, Bevilacqua A (2015). Cancer multicellular spheroids: volume assessment from a single 2D projection. Comput Methods Programs Biomed.

[50] Liu H, Wei Z, Dominguez A, Li Y, Wang X, Qi LS (2015). CRISPR-ERA: a comprehensive designer tool for CRISPR genome editing, (gene) repression, and activation. Bioinformatics.

[51] Zhu G, Zhao XM, Wu J (2016). A survey on biomarker identification based on molecular networks. Quant Biol.

[52] May GHW, Allen KE, Clark W, Funk M, Gillespie DAF (1998). Analysis of the interaction between c-Jun and c-Jun N-terminal kinase in vivo. J Biol Chem.

[53] Yeh AH, Bohula EA, Macaulay VM (2006). Human melanoma cells expressing V600E B-RAF are susceptible to IGF1R targeting by small interfering RNAs. Oncogene.

[54] Cooper J, Giancotti FG (2014). Molecular insights into NF2/Merlin tumor suppressor function. FEBS Lett.

[55] Watanabe K, Montserrat E, Gallinger S, van de Vijver M, López-Bigas N, Bader GD (2010). International network of cancer genome projects. Nature.

[56] Natali PG, Hamby CV, Felding-Habermann B, Liang B, Nicotra MR, Fiippo FD (1997). Clinical significance of aV3 integrin and intercellular adhesion molecule-i expression in cutaneous malignant melanoma lesions. Cancer Res.

[57] Liu J, Lichtenberg T, Hoadley KA, Poisson LM, Lazar AJ, Cherniack AD (2018). An integrated TCGA pan-cancer clinical data resource to drive high-quality survival outcome analytics. Cell.

[58] Simbulan-Rosenthal CM, Dougherty R, Vakili S, Ferraro AM, Kuo LW, Alobaidi R (2019). CRISPR-Cas9 knockdown and induced expression of CD133 reveal essential roles in melanoma invasion and metastasis. Cancers.

[59] Siddique HR, Saleem M (2012). Role of BMI1, a stem cell factor, in cancer recurrence and chemoresistance: preclinical and clinical evidences. Stem Cells.

[60] Jaks V, Barker N, Kasper M, Van Es JH, Snippert HJ, Clevers H (2008). Lgr5 marks cycling, yet long-lived, hair follicle stem cells. Nat Genet.

[61] Da Silva-Diz V, Solé-Sánchez S, Valdés-Gutiérrez A, Urpí M, Riba-Artés D, Penin RM (2012). Progeny of Lgr5-expressing hair follicle stem cell contributes to papillomavirus-induced tumor development in epidermis. Oncogene.

[62] Eguchi R, Wakabayashi I (2020). HDGF enhances VEGF-dependent angiogenesis and FGF-2 is a VEGF-independent angiogenic factor in non-small cell lung cancer. Oncol Rep.

[63] Ke Y, Zhao W, Xiong J, Cao R (2013). Downregulation of miR-16 promotes growth and motility by targeting HDGF in non-small cell lung cancer cells. FEBS Lett.

[64] Bao C, Wang J, Ma W, Wang X, Cheng Y (2014). HDGF: a novel jack-of-all-trades in cancer. Futur Oncol.

[65] Basu-Roy U, Seo E, Ramanathapuram L, Rapp TB, Perry JA, Orkin SH (2012). Sox2 maintains self renewal of tumor-initiating cells in osteosarcomas. Oncogene.

[66] Boumahdi S, Driessens G, Lapouge G, Rorive S, Nassar D, Le Mercier M (2014). SOX2 controls tumour initiation and cancer stem-cell functions in squamous-cell carcinoma. Nature.

[67] Lee SH, Wottrich S, Bonavida B (2017). Crosstalks between Raf-kinase inhibitor protein and cancer stem cell transcription factors (Oct4, KLF4, Sox2, Nanog). Tumor Biol.

[68] Pang L, Xu L, Yuan C, Li X, Zhang X, Wang W (2019). Activation of EGFR-KLF4 positive feedback loop results in acquired resistance to sorafenib in hepatocellular carcinoma. Mol Carcinog.

[69] Riverso M, Montagnani V, Stecca B (2017). KLF4 is regulated by RAS/RAF/MEK/ERK signaling through E2F1 and promotes melanoma cell growth. Oncogene.

[70] Utikal J, Maherali N, Kulalert W, Hochedlinger K (2009). Sox2 is dispensable for the reprogramming of melanocytes and melanoma cells into induced pluripotent stem cells. J Cell Sci.

[71] Yu F, Li J, Chen H, Fu J, Ray S, Huang S (2011). Kruppel-like factor 4 (KLF4) is required for maintenance of breast cancer stem cells and for cell migration and invasion. Oncogene.

[72] Lim ST, Chen XL, Lim Y, Hanson DA, Vo TT, Howerton K (2008). Nuclear FAK promotes cell proliferation and survival through FERM-enhanced p53 degradation. Mol Cell.

[73] Krupkova O, Loja T, Zambo I, Veselska R (2010). Nestin expression in human tumors and tumor cell lines. Neoplasma.

[74] Qin Q, Sun Y, Fei M, Zhang J, Jia Y, Gu M (2012). Expression of putative stem marker nestin and CD133 in advanced serous ovarian cancer. Neoplasma.

[75] Walker L, Millena AC, Strong N, Khan SA (2013). Expression of TGFβ3 and its effects on migratory and invasive behavior of prostate cancer cells: involvement of PI3-kinase/AKT signaling pathway. Clin Exp Metastasis.

[76] Mnich CD, Hoek KS, Oberholzer PA, Seifert B, Hafner J, Dummer R (2007). Reduced pSmad2 immunodetection correlates with increased primary melanoma thickness. Melanoma Res.

[77] Smith MP, Brunton H, Rowling EJ, Ferguson J, Arozarena I, Miskolczi Z (2016). Inhibiting drivers of non-mutational drug tolerance is a salvage strategy for targeted melanoma therapy. Cancer Cell.

[78] Snippert HJ, van der Flier LG, Sato T, van Es JH, van den Born M, Kroon-Veenboer C (2010). Intestinal crypt homeostasis results from neutral competition between symmetrically dividing Lgr5 stem cells. Cell.

[79] Allen EM Van, Wagle N, Sucker A, Treacy DJ, Johannessen CM, Goetz EM (2013). The genetic landscape of clinical resistance to RAF inhibition in metastatic melanoma. Cancer Discov.

[80] Romano E, Pradervand S, Paillusson A, Weber J, Harshman K, Muehlethaler K (2013). Identification of multiple mechanisms of resistance to vemurafenib in a patient with BRAFV600E-mutated cutaneous melanoma successfully rechallenged after progression. Clin Cancer Res.

[81] Li R, Chen T, Li S (2015). Network-based method to infer the contributions of proteins to the etiology of drug side effects. Quant Biol.

[82] Carlino MS, Gowrishankar K, Saunders CAB, Pupo GM, Snoyman S, Zhang XD (2013). Antiproliferative effects of continued mitogen-activated protein kinase pathway inhibition following acquired resistance to BRAF and/or MEK inhibition in melanoma. Mol Cancer Ther.

[83] Long GV, Fung C, Menzies AM, Pupo GM, Carlino MS, Hyman J (2014). Increased MAPK reactivation in early resistance to dabrafenib/trametinib combination therapy of BRAF-mutant metastatic melanoma. Nat Commun.

[84] Greco SJ, Liu K, Rameshwar P (2007). Functional similarities among genes regulated by OCT4 in human mesenchymal and embryonic stem cells. Stem Cells.

[85] Angelastro JM, Lamé MW (2010). Overexpression of CD133 promotes drug resistance in C6 glioma cells. Mol Cancer Res.

[86] Siddique HR, Saleem M (2012). Concise review: role of BMI1, a stem cell factor, in cancer recurrence and chemoresistance: preclinical and clinical evidences. Stem Cells.

[87] Ryuge S, Sato Y, Jiang SX, Wang G, Kobayashi M, Nagashio R (2013). The clinicopathological significance of Lgr5 expression in lung adenocarcinoma. Lung Cancer.

[88] Sánchez-Danés A, Larsimont JC, Liagre M, Muñoz-Couselo E, Lapouge G, Brisebarre A (2018). A slow-cycling LGR5 tumour population mediates basal cell carcinoma relapse after therapy. Nature.

[89] Yang L, Xie X, Tang H, Kong Y, Xie X, Chen J (2015). LGR5 promotes breast cancer progression and maintains stem-like cells through activation of wnt/β-catenin signaling. Stem Cells.

[90] de Lau W, Peng WC, Gros P, Clevers H (2014). The R-spondin/Lgr5/Rnf43 module: regulator of Wnt signal strength. Genes Dev.

[91] Chen X, Yun J, Fei F, Yi J, Tian R, Li S (2012). Prognostic value of nuclear hepatoma-derived growth factor (HDGF) localization in patients with breast cancer. Pathol Res Pract.

[92] Kishima Y, Yamamoto H, Izumoto Y, Yoshida K, Enomoto H, Yamamoto M (2002). Hepatoma-derived growth factor stimulates cell growth after translocation to the nucleus by nuclear localization signals. J Biol Chem.

