# Supplementary material for: Systematic screening reveals synergistic interactions that overcome MAPK inhibitor resistance in cancer cells
Source: Cancer Biol Med. 2021 Jun 9;19(2):229–52. doi: 10.20892/j.issn.2095-3941.2020.0560 (PMC8832956; doi:10.20892/j.issn.2095-3941.2020.0560)
Supplement: Supplementary file 1 [file cbm-19-229-s001.pdf]

Supplementary materials

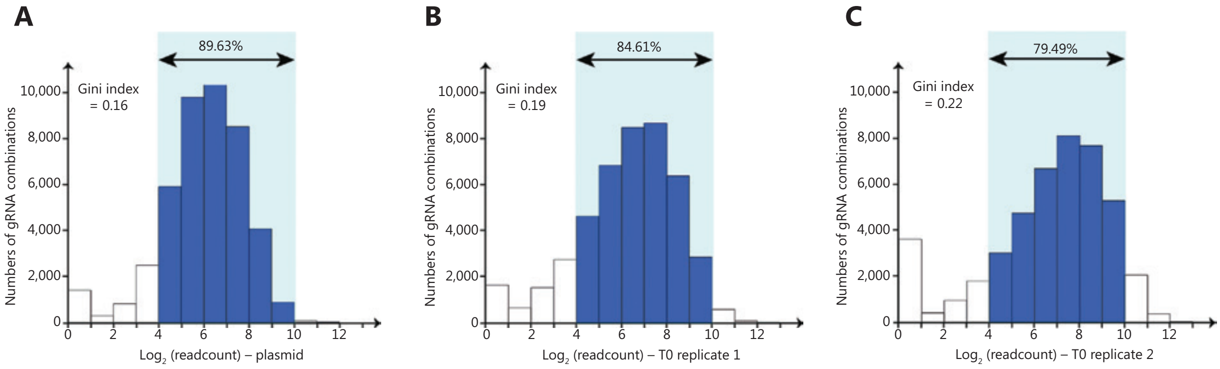

Figure S1 Histogram of pair-wise sgRNA combinations in different libraries. (A–C) Distributions of pair-wise sgRNA combinations in library samples after plasmid preparation (A) and after lentiviral infection and puromycin selection in 2 replicates (B, C). The Gini coefficients were calculated to evaluate the distribution of sgRNA combinations. The shaded areas represent the maximal coverage of sgRNA combinations within a 64-fold range in plasmid samples, and 2 T0 replicates, respectively.

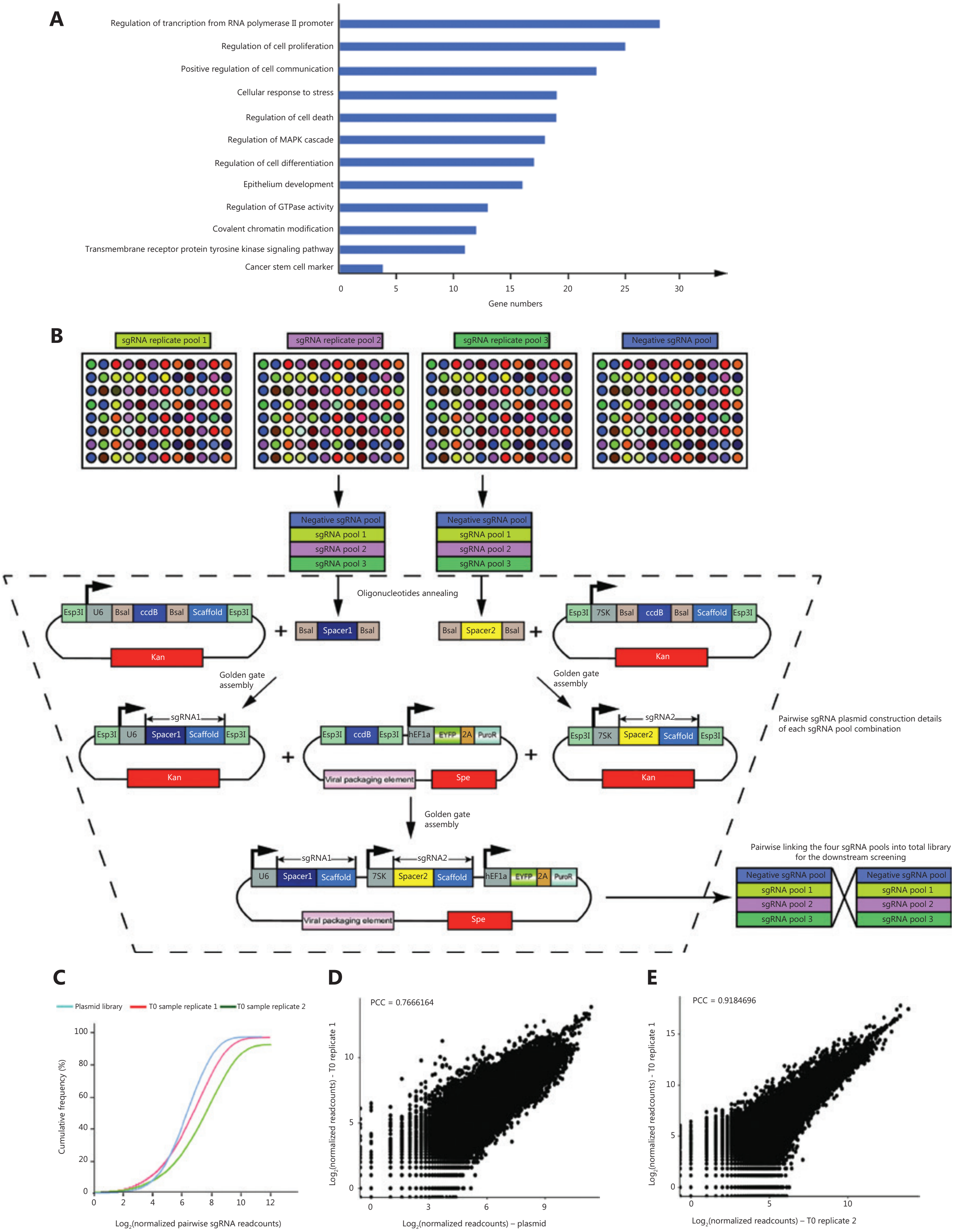

Figure S2 Construction of the pair-wise sgRNA library. (A) Genes were classified into functional groups according to the gene ontology information from the Molecular Signature Database. (B) Workflow of pair-wise sgRNA library construction. Synthesized and annealed oligos

were mixed and cloned into the single-wise sgRNA constructs in the first Golden Gate reaction with BsaI. The single-wise sgRNA constructs were cloned into a lentiviral vector in the second Golden Gate reaction with Esp3I. Kan, kanamycin resistance gene; Spe, spectinomycin resistance gene; ccdB, ccdB toxin coding gene; PuroR, puromycin resistance gene. (C) Cumulative distribution of normalized pair-wise sgRNA read counts in the indicated sgRNA libraries. The cumulative coverages of the plasmid library (blue line), and two independent replicated samples before drug treatment (red and green lines) were 97.26%, 97.25%, 92.76%, respectively. (D, E) Pearson's correlations of the indicated samples.

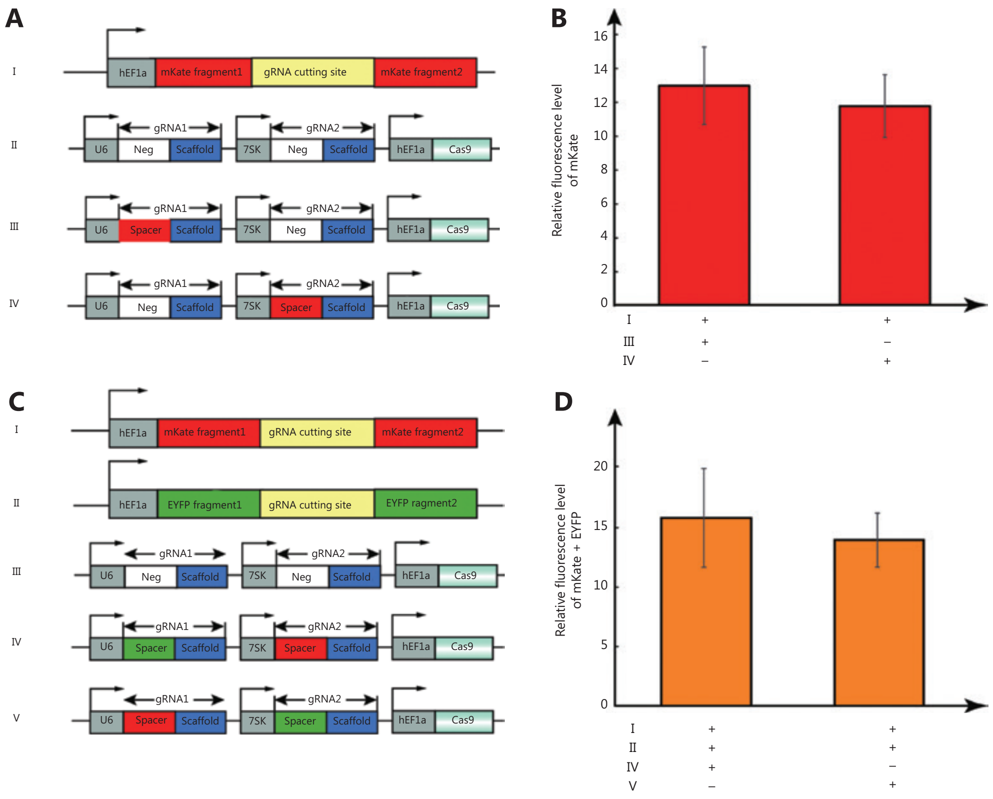

Figure S3 Balancing test of pair-wise promoter configurations. (A) Plasmids used for cutting insulating linker to catalyze the recombination of mKate fragments. (B) The relative fluorescence expression level of mKate, which was calculated from the induced fluorescence level of vector III or IV divided by the leaky fluorescence level of the negative vector II. The bar plot is the mean ± s.d. (N = 3) from 3 independent replicates. There was no significant difference between U6 initiated relative fluorescence and 7SK initiated relative fluorescence according to the unpaired 2-tailed Student's t-test. (C) Plasmids used for cutting insulating linker to catalyze recombination of mKate and EYFP fragments. (D) Relative fluorescence level of mKate + EYFP was calculated from the induced fluorescence level of vector IV or V divided by the leaky fluorescence level of the negative vector III. Bar plot was the mean ± s.d. (N = 3) from 3 independent replicates. There was no significant difference between U6 initiated relative fluorescence and 7SK initiated relative fluorescence according to the unpaired 2-tailed Student's t-test.

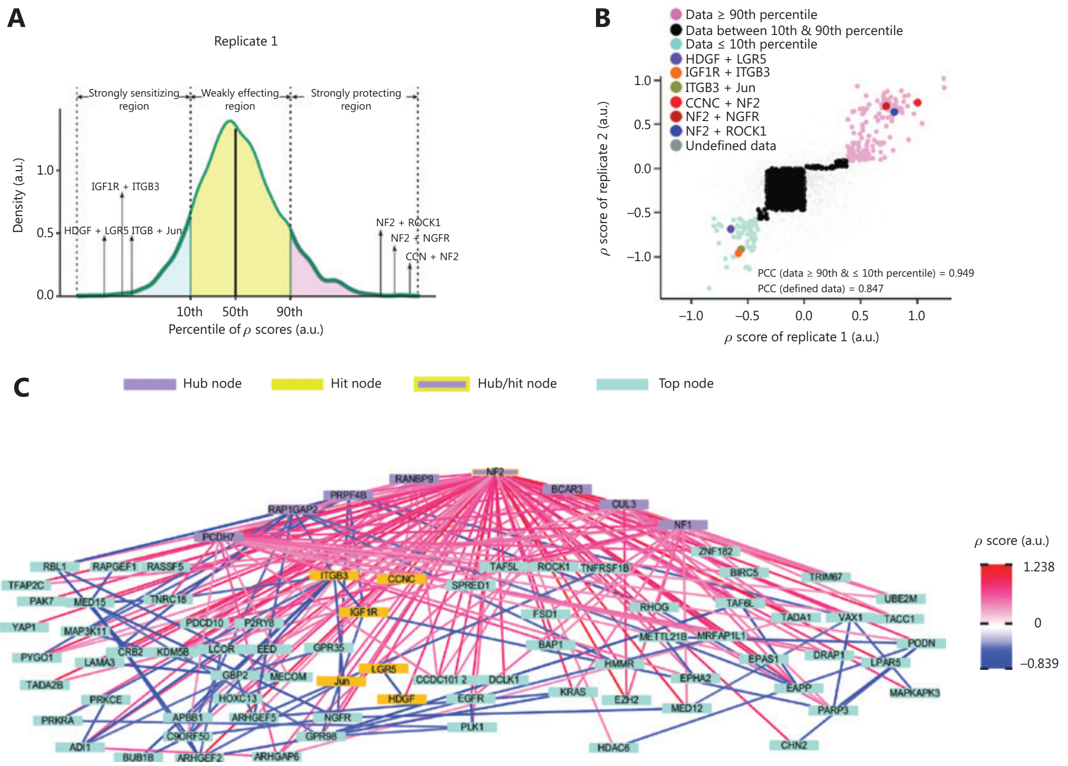

Figure S4 Identification of gene pairs with strong growth phenotypes during vemurafenib stress. (A) Histogram distribution of ρ scores in replicate 1. The ρ scores below the 10th percentile or above the 90th percentile were recognized as gene pairs with strong growth phenotype. (B) Correlation of ρ scores of 2 independent replicates. Blue and pink points represent the reproducible top and bottom 10% of ρ scores; black points indicate the middle 80% reproducible ρ scores; and gray points represent the other ρ scores that showed a low correlation in 2 independent replicates. (C) The hierarchical network representation of the top listed effective gene pairs **(Supplementary Table S4)**. The thickness of edges indicates the strength of ρ scores. Blue and red colors designate the sensitizing and protective growth phenotypes, respectively. Purple nodes inside yellow rectangles denote hub gene nodes. Blue nodes represent top gene nodes. Yellow nodes were selected for the experimental validations shown in subsequent figures.

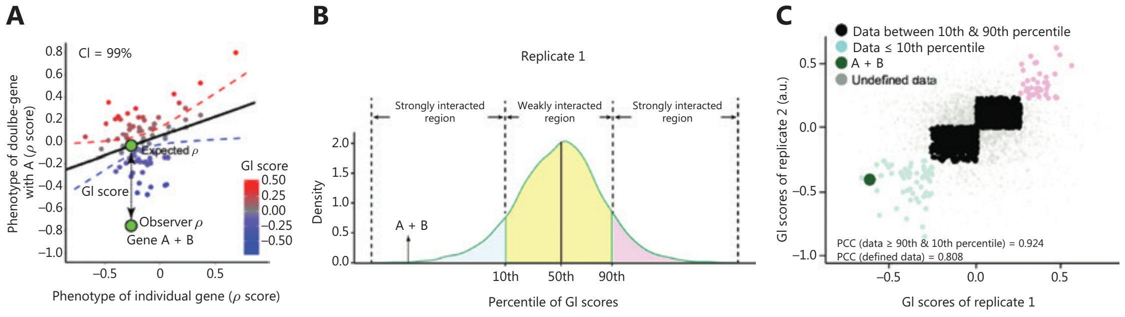

Figure S5 The strategy for selecting strong genetic interactions. (A) Scatter plot of ρ scores of pair-wise sgRNA perturbations against a common bait gene (for example, *Gene A*) and a series of single-wise sgRNA perturbations. *GI* scores were obtained by calculating the deviations between observed and expected ρ scores. The dotted line denotes the 99% confidence interval). (B) The histogram distribution of averaged *GI* scores for targeting the same gene pair with both sgRNA orientations derived from replicate 1. *GI* scores below the 10th percentile or above the 90th percentile were recognized as strong interactions. (C) The correlation of *GI* scores of 2 independent replicates. Blue and pink points represent the reproducible top and bottom 10% of *GI* scores; black points indicate the middle 80% reproducible *GI* scores; and gray points represented the undefined *GI* scores that showed a low correlation in 2 independent replicates.

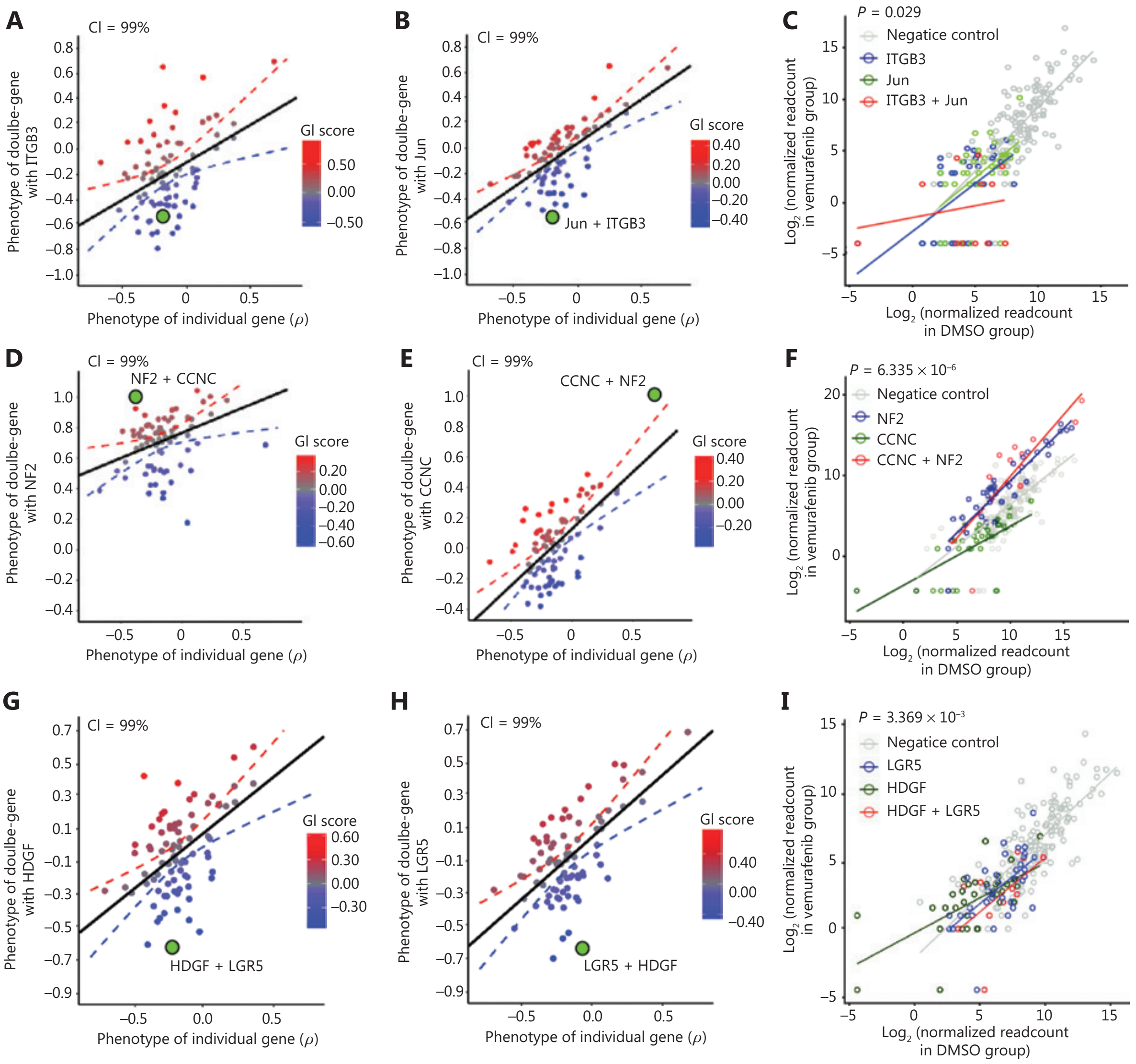

Figure S6 Validation of identified genetic interactions. (A, B), (D, E), (G, H) Scatter plots of ρ scores of pairwise sgRNA perturbations against a common bait gene and single-wise sgRNA perturbations. *GI* scores were calculated as the deviation from the observed ρ scores to linearly fitted values (black line). The dotted line denotes the 99% confidence interval. The color bar represents *GI* scores from negative (blue) to positive (red) effects. (C, F and I) Scatter plots of the read count abundances of single-wise and pair-wise sgRNA species in the vemurafenib- and dimethyl sulfoxide-treated samples. Red dots, pair-wise sgRNA species; green and blue, single-wise sgRNA species; and gray dots, negative control sgRNA. Linear fit lines for pair-wise (red), single-wise (green, blue), and negative control (gray) sgRNA species. Data were tested for significance using the 2-tailed Mann-Whitney U test. (C, F and I) The *P* values show the results of Mann-Whitney U tests when comparing the distribution of ρ scores perturbing the gene pairs against the negative controls.

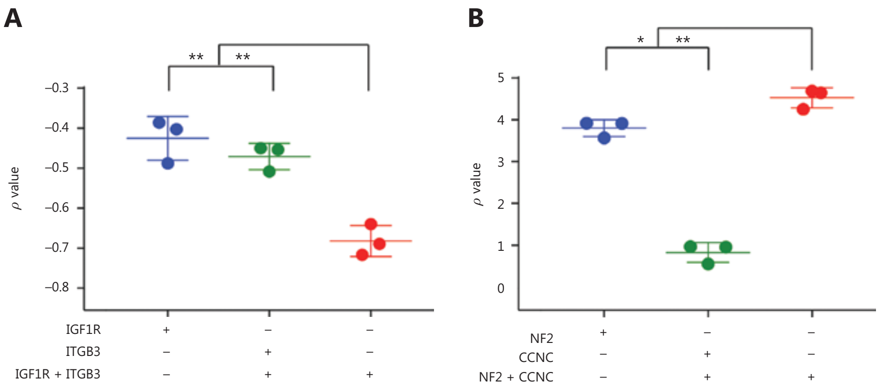

Figure S7 The sgRNA growth competition assay for GI pairs. (A, B) Schematic gene perturbations were conducted with lentiviral sgRNA expression vectors, which were different from those in Figure 3 (E, G). Mixed Cas9-A375 cells with or without single or double gene perturbations were treated with vemurafenib or dimethyl sulfoxide for up to 10 days before fluorescence-activated cell sorting. The ρ values were calculated by using the relative abundances of individual Cas9-A375 derivatives (see details in the Materials and Methods). Data are the mean ± s.d. (N = 3) from 3 independent replicates. "*" and "**" designate significant differences (P < 0.05 and P < 0.01, respectively) between single and pair-wise sgRNA perturbations according to the unpaired 2-tailed Student's t-test.

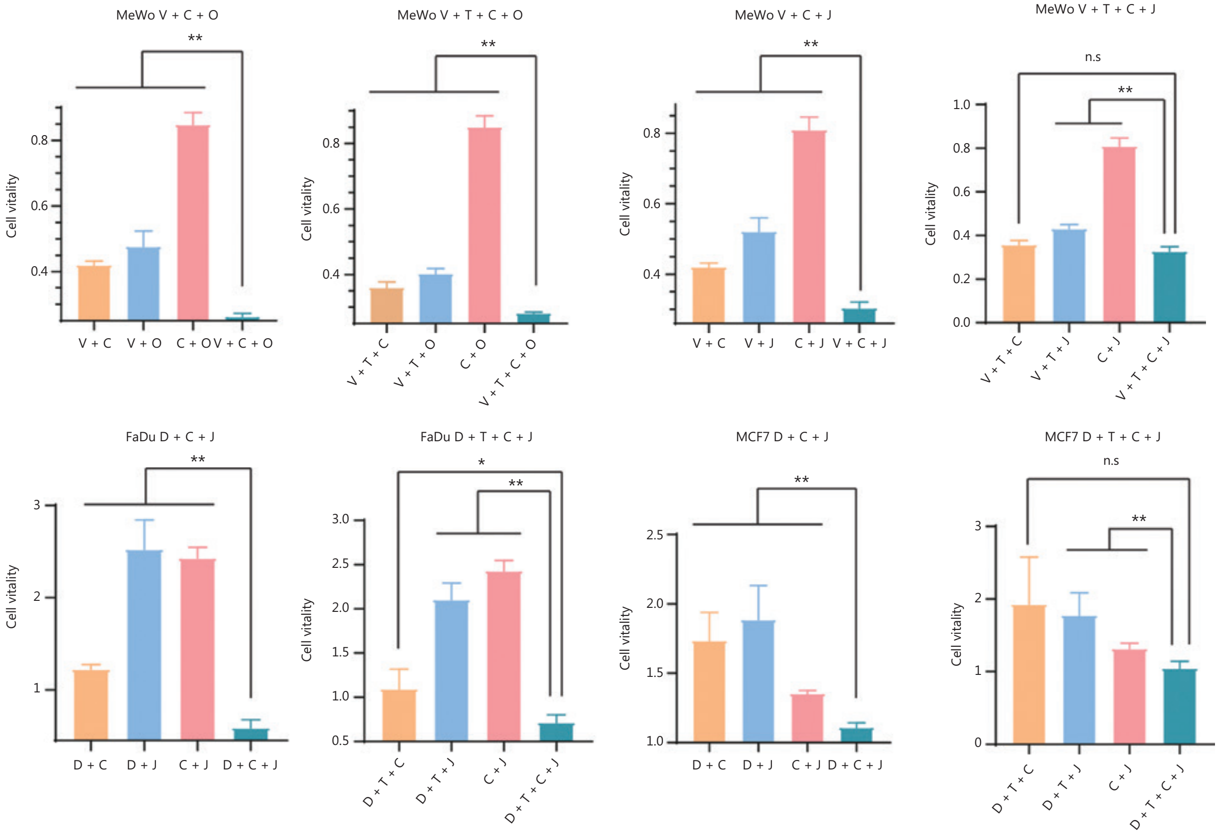

Figure S8 Cell viability tests of the MAPKi sensitizing effect by using different drug combinations. MeWo, FaDu, and MCF7 cells were used as models to validate the efficiency of MAPKi sensitizing effects when using screening selected combinatorial hits. V, D, C, O, J were abbreviated as vemurafenib, dabrafenib, cilengitide, OSI-096 (linsitinib), and JNK-IN-8, respectively. Bar plots denote the mean ± s.d. (N = 3) from 3 independent replicates, and the unpaired 2-tailed Student's t-test was used to evaluate the statistical significance, "*" denotes P < 0.05, "**" denotes P < 0.01.

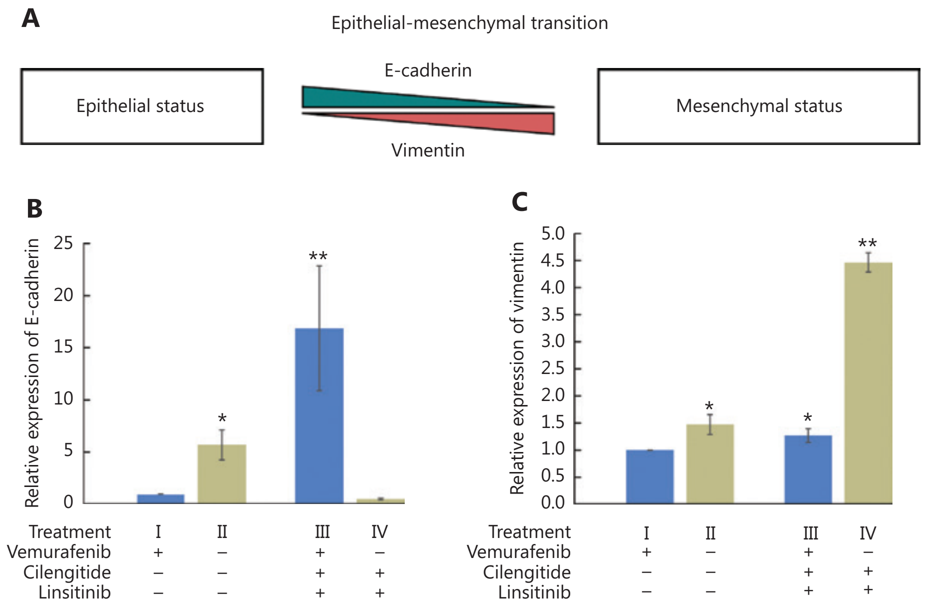

Figure S9 Gene expressions of E-cadherin and vimentin are affected by different combinatorial drug regimens. (A) Schematic illustration of E-cadherin and vimentin gene expressions as significant biomarkers of the epithelial-mesenchymal transition. (B) The mRNA expressions of E-cadherin in different portfolio treatments of clilengitide + linsitinib. (C) The mRNA expressions of vimentin in the combinatorial treatments of clilengitide + linsitinib. "*" and "**" designate significant differences ($P < 0.05$ and $P < 0.01$, respectively) between single drug vemurafenib *vs.* combinatorial drug treatments tested using the unpaired 2-tailed Student's *t*-test.

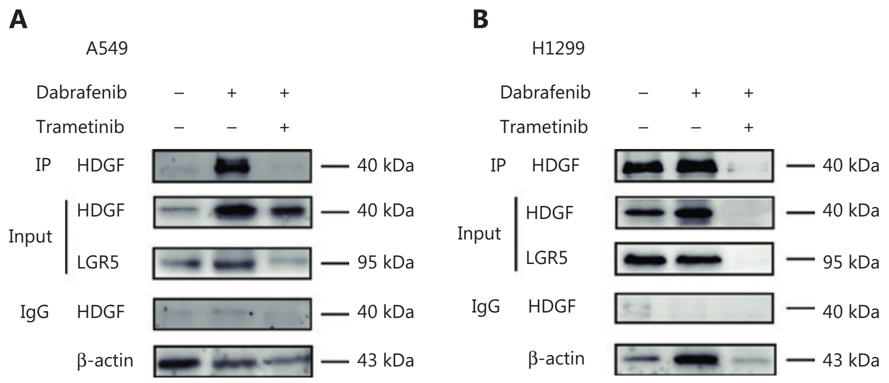

Figure S10 HDGF and LGR5 form a protein complex to respond to MAPKi stress. (A) Immunoprecipitation assays (IP) with anti-LGR5 antibody extracts from A549 cells expressing endogenous HDGF exposed to dimethyl sulfoxide (DMSO), BRAFi, or BRAFi + MEKi for 72 h. (B) IP assays with anti-LGR5 antibody extracts from H1299 cells expressing endogenous HDGF exposed to DMSO, BRAFi, or BRAFi + MEKi for 72 h.

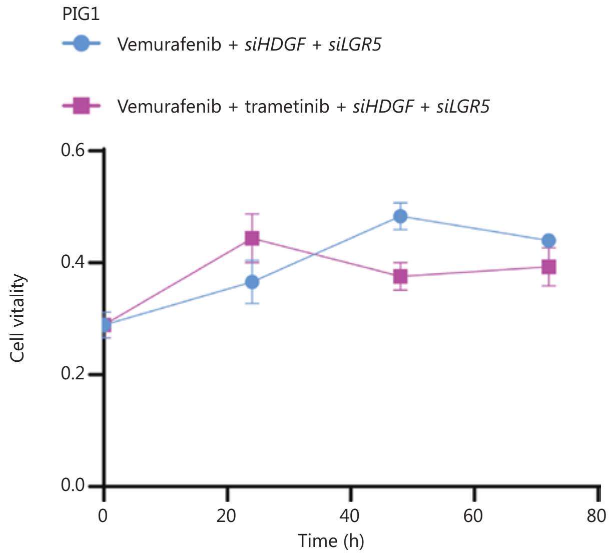

Figure S11 The CCK8 assay for detecting PIG1 cell growth. The cell viability assay showing the growth curve of PIG1 cells during treatments with vemurafenib + *siHDGF* + *siLGR5* and vemurafenib + trametinib + *siHDGF* + *siLGR5*.

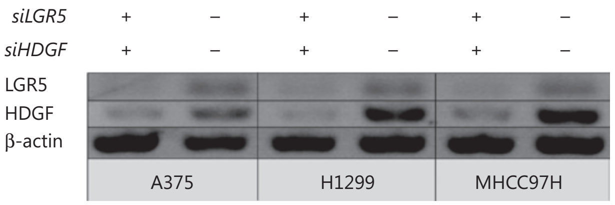

Figure S12 The *siHDGF* and *siLGR5* co-knockdown efficiency in A375, H1299, and MHCC97H cell lines. The *HDGF* and *LGR5* co-knockdown efficiency in A375, H1299, and MHCC97H cells. A375, H1299, and MHCC97H cells were simultaneously co-transfected with *siHDGF* and *siLGR5* in the experimental groups for 48 h.

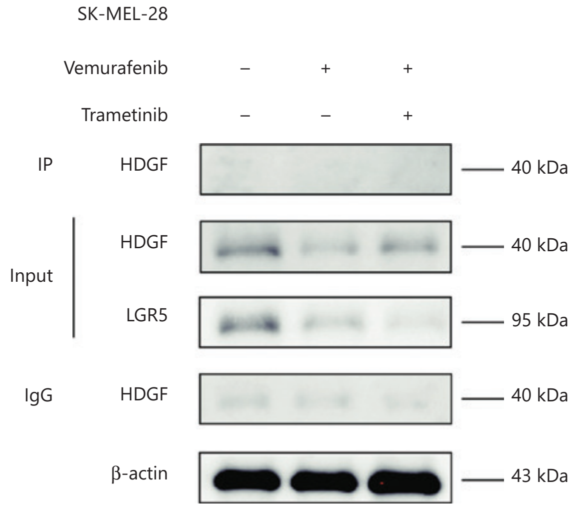

Figure S13 HDGF and LGR5 cannot form a protein complex during MAPKi stress. Immunoprecipitation assays with anti-LGR5 antibody extracts from SK-MEL-28 cells expressing endogenous HDGF exposed to dimethyl sulfoxide, BRAFi, or BRAFi + MEKi for 72 h.

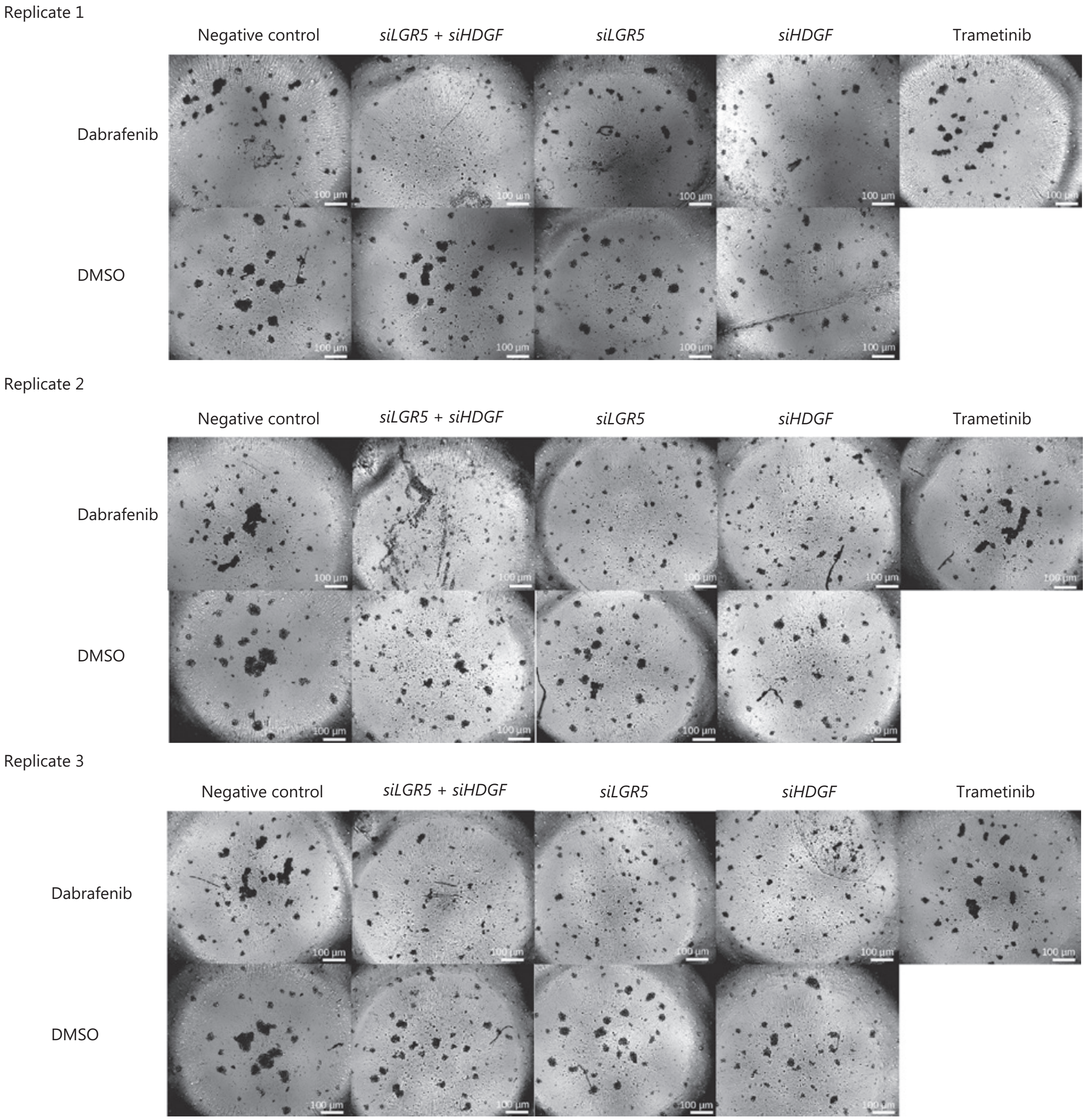

Figure S14 Images of MHCC97H spheroids during perturbations by different drug combinations. The images were captured using a 4× microscope objective, from 3 independent replicates. Scale bars, 1,000 μm (white).

Table S1 Information of sgRNAs used in the efficiency tests of 2 promoters

Plasmids used in Supplementary Figure S3A	sgRNA sequences
U6-mKate_linker_sgRNA-7SK-negative_sgRNA-EF1α-spCas9	ACGGAGGCTAAGCGTCGCAA<br>ACGTTCGAGTACGACCAGCT
U6-negative_sgRNA-7SK-mKate_linker_sgRNA-EF1α-spCas9	ACGTTCGAGTACGACCAGCT<br>ACGGAGGCTAAGCGTCGCAA
U6-negative_sgRNA-7SK-negative_sgRNA-EF1α-spCas9	ACGTTCGAGTACGACCAGCT<br>ACGTTCGAGTACGACCAGCT
**Plasmid used in Supplementary Figure S3C**	**sgRNA sequences**
U6-mKate_linker_sgRNA-7SK-EYFP_linker_sgRNA-EF1α-spCas9	ACGGAGGCTAAGCGTCGCAA<br>TACTAACGCCGCTCCTACAG
U6-EYFP_linker_sgRNA-7SK-mKate_linker_sgRNA-EF1α-spCas9	TACTAACGCCGCTCCTACAG<br>ACGGAGGCTAAGCGTCGCAA
U6-negative_sgRNA-7SK-negative_sgRNA-EF1α-spCas9	ACGTTCGAGTACGACCAGCT<br>ACGTTCGAGTACGACCAGCT

Table S2 Information of sgRNAs used in the growth competition assay

Plasmid DNA used in Figure 2E	sgRNA sequence
pLV-hU6-gRNA-neg-7sk-IGF1R-hEF1a-EYFP-2A-Puro	GATCCTCACGGTGAACGTCT<br>CTTCGAGATGACCAATCTCA
pLV-hU6-gRNA-neg-7sk-ITGB3-hEF1a-EBFP-2A-Puro	GATCCTCACGGTGAACGTCT<br>CTGGCGGGCGTTGGCGTAGG
pLV-hU6-IGF1R-7sk-ITGB3-hEF1a-mKate-2A-Puro	CTTCGAGATGACCAA TCTCA<br>CTGGCGGGCGTTGGCGTAGG
**Plasmid DNA used in Figure 2F**	**sgRNA sequence**
pLV-hU6-gRNA-neg-7sk-CCNC-hEF1a-EYFP-2A-Puro	GATCCTCACGGTGAACGTCT<br>TAGGCAAAGATCCGTTCTGT
pLV-hU6-gRNA-neg-7sk-NF2-hEF1a-EBFP2-2A-Puro	GATCCTCACGGTGAACGTCT<br>ATCCTCACGGTGAACGTCTT
pLV-hU6-CCNC-7sk-NF2-hEF1a-mKate-2A-Puro	TAGGCAAAGATCCGTTCTGT<br>ATCCTCACGGTGAACGTCTT
**Plasmid DNA used in Supplementary Figure S7A**	**sgRNA sequence**
pLV-hU6-gRNA-neg-7sk-IGF1R-hEF1a-EBFP-2A-Puro	ATTTGCCGTTCGAATGCGTA<br>GCTCTCGAGGCCAGCCACT
pLV-hU6-gRNA-neg-7sk-ITGB3-hEF1a-EYFP-2A-Puro	ATTTGCCGTTCGAATGCGTA<br>AGTGAGGCCCGAGTACTAG
pLV-hU6-IGF1R-7sk-ITGB3-hEF1a-mKate-2A-Puro	GCTCTCGAGGCCAGCCACT<br>AGTGAGGCCCGAGTACTAG
**Plasmid DNA used in Supplementary Figure S7B**	**sgRNA sequence**
pLV-hU6-gRNA-neg-7sk-CCNC-hEF1a-EYFP-2A-Puro	ATTTGCCGTTCGAATGCGTA<br>GATGCCAAAAACACACATGT
pLV-hU6-gRNA-neg-7sk-NF2-hEF1a-EBFP2-2A-Puro	ATTTGCCGTTCGAATGCGTA<br>GATCCTCACGGTGAACGTCT
pLV-hU6-CCNC-7sk-NF2-hEF1a-mKate-2A-Puro	GATGCCAAAAACACACATGT<br>GATCCTCACGGTGAACGTCT

Table S3 List of previously reported genes involved in vemurafenib resistance

Gene	Supporting literature
BAP1	Carbone M, Yang H, Pass HI, Krausz T, Testa JR, Gaudino G. BAP1 and cancer. Nat Rev Cancer. 2013; 13: 153–159.
BIRC5	Ji Z, Kumar R, Taylor M, Rajadurai A, Marzuka-Alcala A, Chen YE, et al. Vemurafenib Synergizes with Nutlin-3 to Deplete Survivin and Suppresses Melanoma Viability and Tumor Growth. Clin Cancer Res. 2013; 19: 4383–4391.
DCLK1	Nakanishi Y, Seno H, Fukuoka A, Ueo T, Yamaga Y, Maruno T, et al. Dclk1 distinguishes between tumor and normal stem cells in the intestine. Nat Genet. 2012; 45: 98–103.
EPHA2	Miao B, Ji Z, Tan L, Taylor M, Zhang J, Choi HG, et al. EPHA2 Is a Mediator of Vemurafenib Resistance and a Novel Therapeutic Target in Melanoma. Cancer Discov. 2014; 5: 274–287.
EZH2	Zingg D, Julien Debbache, Schaefer SM, Tuncer1 E, Frommel SC, Cheng P, et al. The epigenetic modifier EZH2 controls melanoma growth and metastasis through silencing of distinct tumour suppressors. Nat Commun. 2015; 6: 1–17.
HDAC6	PENG U, WANG Z, PEI S, OU Y, HU P, LIU W, et al. ACY-1215 accelerates vemurafenib induced cell death of BRAF-mutant melanoma cells *via* induction of ER stress and inhibition of ERK activation. Oncol Rep. 2017; 37: 1270–1276.
KDM5B	Roesch A, Vultur A, Bogeski I, Wang H, Zimmermann KM, Speicher D, et al. Overcoming Intrinsic Multidrug Resistance in Melanoma by Blocking the Mitochondrial Respiratory Chain of Slow-Cycling JARID1Bhigh Cells. Cancer Cell. 2013; 23: 811–825.
NGFR	Lehraiki A, Abbe P, Cerezo M, Rouaud F, Allegra M, Kluza J, et al. Increased CD271 expression by the NF-kB pathway promotes melanoma cell survival and drives acquired resistance to BRAF inhibitor vemurafenib. Cell Discov. 2015; 1: 1–13.
ROCK1	Smit MA, Maddalo G, Greig K, Raaijmakers LM, Possik PA, Breukelen B van, et al. ROCK1 is a potential combinatorial drug target for BRAF mutant melanoma. Mol Syst Biol. 2014; 10: 772–772.
YAP1	Kim MH, Kim J, Hong H, Lee S-H, Lee J-K, Jung E, et al. Actin remodeling confers BRAF inhibitor resistance to melanoma cells through YAP/TAZ activation. EMBO J. 2016; 35: 462–478.

Table S4 Information of 84 selected genes and designed sgRNAs

Gene	sgRNA	Function category
ADI1	TGGTAGTTTATCTTTGCATA	/
	AGACATGGTGACGCTCCCCG	
	TATGGTTATGATGTCCATCC	
APBB1	AAGGTCAACGTCCCCTCCTC	Regulation of transcription from RNA polymerase II promoter; regulation of cell death; regulation of cell differentiation; covalent chromatin modification
	AGGCTGGGTAGAGATGACCG	
	ATTACTGGCACATCCCAACA	
ARHGAP6	GAATGGTGATCTGACAGCTC	Regulation of GTPase activity
	CTCGGAATATCCCCACTGTC	
	GAAACTGGATTCACTAGGAA	
ARHGEF2	CAAGGAGGAGCGGCCACGGC	Regulation of transcription from RNA polymerase II promoter; regulation of cell proliferation; regulation of cell death; regulation of cell differentiation; regulation of GTPase activity
	GCTATTAGAACGCCGACGCC	
	TTTGGGCCCGAGGGTCCATG	
ARHGEF5	CCTCCGAGTCGTACCTGCAG	Regulation of transferase activity; regulation of MAPK cascade; regulation of GTPase activity
	CCGCTGCAGGTACGACTCGG	
	CACGGTGCTGTTGCGCACCA	

Table S4 Continued

Gene	sgRNA	Function category
BAP1	GCTCTTCGATCCATTTGAAC<br>CACGGACGTATCATCCACCA<br>CTTCCACGAGCAGGGTGAAG	Regulation of cell proliferation; covalent chromatin modification
BCAR3	TCGGCAGGTAGCTCTCTGAC<br>AATGGCCATCGGCATTGCAG<br>GAAGGAATAGAGGTCCCCCA	Regulation of GTPase activity
BIRC5	GCCAGGCAGGGGGCAACGTC<br>GCTCGTTCTCAGTGGGGCAG<br>CACTGAGAACGAGCCAGACT	Regulation of cell death
BUB1B	ACAGTTACTTGCACAACCAA<br>ATGAGCTGCAAGCAGGCCCT<br>GCTCCAATCATCCGTGTAGG	Regulation of transferase activity
C9orf50	AATACACCCCTTTGATGTTG<br>CTGCCTGGGCGCCGATTCCC<br>CCGCCGGGTCGCGGTGAGCA	/
CCDC101	GCGGGAATCGGCAGACACGA<br>GCATCCGCTCATGGGTCTTC<br>GGGTTTGTTTGATCAGCTGA	Covalent chromatin modification
CCNC	GTACAACACAGGCTACATGT<br>GATGCCAAAAACACACATGT<br>TAGGCAAAGATCCGTTCTGT	Regulation of transcription from RNA polymerase II promoter; regulation of transferase activity
CHN2	CAAATGATTCTCTTGGGACG<br>AGGACACCGACGAGCCGGAC<br>GTGGATCATTAGGTACCGGA	Regulation of GTPase activity
CRB2	TGTGGGCCCATGGAGCCCCG<br>ACATCGATGAGTGTGCATCC<br>TGCCAGGCTACCGAGAGTGG	Regulation of transcription from RNA polymerase II promoter; regulation of cell differentiation; epithelium development
CUL3	GAGATCAAGTTGTACGTTAT<br>TGGATATGATTGCAAGAGAG<br>CGAGATCAAGTTGTACGTTA	Regulation of transcription from RNA polymerase II promoter; regulation of cell proliferation; regulation of transferase activity
DCLK1	GGAGTAGAGAGCTGACTACC<br>GAGATACAGCCGAGGGTCGC<br>GCACTTCGACGAGCGGGATA	Cancer Stem cell marker
DRAP1	TGTCGGGAACAGATGCCACC<br>TCCCCGTCCCCCTGCATGTC<br>GGACGGGGAAGACAACCACA	Regulation of transcription from RNA polymerase II promoter;

Table S4 Continued

Gene	sgRNA	Function category
EAPP	GGATGACTACGACCCCTACG<br>CTAGGCTCTTCAACCGCGTA<br>ACTAGATGATTCACTTTCTC	Regulation of transcription from RNA polymerase II promoter; regulation of cell proliferation
EED	GCAGGCATGTCTGTTCCCGC<br>CGCAGTCGACACTTCCCTCT<br>TGTTTGGAGTTCAGTTTAAC	Regulation of transcription from RNA polymerase II promoter; covalent chromatin modification
EGFR	CCCCCGGGAGAGCGACTGCC<br>AATTCGCTCCACTGTGTTGA<br>TGCAAATAAAACCGGACTGA	Regulation of transcription from RNA polymerase II promoter; regulation of cell proliferation; regulation of cell death; regulation of transferase activity; regulation of MAPK cascade; epithelium development; regulation of GTPase activity
EPAS1	GTCCATCTGCTGGTCAGCTT<br>GAAAGATCATGTCGCCATCT<br>CTGCTGGTCAGCTTCGGCTT	Regulation of transcription from RNA polymerase II promoter;
EphA2	CCACACAGGCACCGATATCC<br>GTGTGCAAGGCATCGACGCT<br>CTACTATGCCGAGTCGGACC	Regulation of MAPK cascade; epithelium development; regulation of GTPase activity
EZH2	GGAAGCAGGGACTGAAACGG<br>GGGAAGCAGGGACTGAAACG<br>ACACGCTTCCGCCAACAAAC	Regulation of transcription from RNA polymerase II promoter; regulation of cell proliferation; regulation of transferase activity; regulation of MAPK cascade; regulation of cell differentiation; regulation of GTPase activity; covalent chromatin modification
FSD1	CAGCCGTACCTACGAGCTGC<br>ACAGATGCTGCTGAACGTGG<br>CTGCAGCTCGTAGGTACGGC	/
GAB2	CTGTTGGCCATGGAACGAGC<br>ATACTCCTGAGGTGCGCTGG<br>TGCGCTCTCGGAGAAGGTGC	Regulation of cell proliferation
GBP2	AACTTTCGGATGCACAACCG<br>CTCAGTGTCGAGCAGAACTA<br>GGAAGTGCTTCGTCTTCGAT	Cancer Stem cell marker
GPR35	TGGTTATGTACAGGGCGCGA<br>TCGCAGGGAGTGCAGCACGA<br>CGGCCGTGTGCGCGGTCCTC	/
GPR98	GGTATACAGGGGCTCCCCCA<br>CTGAAATCTAGATTCAGGCG<br>GATGTCATCCAGAAACACTG	Regulation of cell differentiation
HDAC6	CTCTATCCCCAATCTAGCGG<br>TCCATCCACCGCTACGAGCA<br>ACCTAATCGTGGGACTGCAA	Regulation of cell death; regulation of cell differentiation; covalent chromatin modification

Table S4 Continued

Gene	sgRNA	Function category
HDGF	TAGCCGGAAGCCTTGACAGT	Regulation of transcription from RNA polymerase II promoter;
	GCAAGCCCAACAAGAGGAAA	
	AGCCGGAAGCCTTGACAGTA	
HMMR	TGAAAGGCTGGTCAAGCAAT	/
	TAAAGAGAAGAATCTGTTTG	
	TGCCCAGGACAGGCGGATCC	
HOXC13	GTACGTCTATGAGGACAGCG	Regulation of transcription from RNA polymerase II promoter; epithelium development
	AGTGCTGGTAGCCTTCGACG	
	GACGCCCTCATCCCCGTCGA	
IGF1R	GGCTCTCGAGGCCAGCCACT	Regulation of cell proliferation; regulation of cell death; regulation of transferase activity; regulation of MAPK cascade
	TCGTTGCGGATGTCGATGCC	
	CTTCGAGATGACCAATCTCA	
ITGB3	TTCTCCTTCAGGTCACAGCG	Regulation of transferase activity; regulation of cell differentiation
	GAGTGAGGCCCGAGTACTAG	
	CTGGCGGGCGTTGGCGTAGG	
Jun	GCGGTAGCCTCGGTGGCAGG	Regulation of transcription from RNA polymerase II promoter; regulation of cell proliferation; regulation of cell death; regulation of MAPK cascade; regulation of cell differentiation; epithelium development; regulation of GTPase activity
	CTGCTGGGGTTGCGCGGGAA	
	CAGACAGTGCCCGAGATGCC	
KDM5B	TGCTGAGATGCGTATCCGTT	Regulation of cell proliferation; epithelium development; covalent chromatin modification; cancer Stem cell marker
	CAGTCTGCTCGGCTATGGGC	
	CGGCTATGGGCCGGATCTTG	
KRAS	CGAATATGATCCAACAATAG	Regulation of cell proliferation; regulation of cell death; regulation of transferase activity; regulation of MAPK cascade; regulation of cell differentiation; epithelium development
	GTAGTTGGAGCTGGTGGCGT	
	GAATATAAACTTGTGGTAGT	
LAMA3	GCGTTCACAGTGCTCTCCCG	Epithelium development
	GGGAATCCCCAGAAATTCGG	
	GCCCATTGTTCTCCCCCTGG	
LCOR	AGATCCATTCCTCGAACTTG	Regulation of transcription from RNA polymerase II promoter;
	TAATCCAGATGGCCCAAGGC	
	AGTACCGCCCAGACGGACTT	
LGR5	GCTCTGACATACATTCCCAA	Regulation of cell proliferation; epithelium development; cancer Stem cell marker
	CACTGTCATTGCGAGCCCGA	
	CGGAGACTGGGCAGGGGATT	
LPAR5	CCAGAGGGCTAGCGCGTTGA	/
	ACTCGTCGGGCCGAGTCTTC	
	ATCTTCCAGATGAACATGTA	

Table S4 Continued

Gene	sgRNA	Function category
MAP3K11	AGAGCTGCTACCGCGCTCTG	Regulation of cell death; regulation of transferase activity; regulation of MAPK cascade
	CAGCGCCCGCTGGATCAGCT	
	AGCTGCTACCGCGCTCTGCG	
MAPKAPK3	ACTTCTGTCCAGTGCGCCGA	Regulation of transferase activity; regulation of MAPK cascade
	CTTGGACAACTGGTAGTCGT	
	TGTATGACAGCCCCAAGGCC	
MECOM	TTGTTTGAGGCCCGACGAAG	Regulation of cell death; regulation of MAPK cascade; covalent chromatin modification
	GGCCTGTGGTACAAGCCGGA	
	GATGATGTCAGTACACCAAG	
MED12	AGTCCGAGTGGACCGGCGAG	Regulation of transcription from RNA polymerase II promoter; epithelium development
	GATTGCTGCATAGTAGGCAC	
	CCGGCCCCGCGCCTATTACC	
MED15	CCCAGAGCCCAGTGACGGCG	Regulation of transcription from RNA polymerase II promoter;
	GGCACACAGTAAATCCAGCA	
	TCGAAAATGGATAATGAGCC	
METRL21B	AGGCAAGAAGGTGATCGAAC	/
	GAAGGTGATCGAACTGGGTG	
	AGGTGATCGAACTGGGTGCG	
MRFAP1L1	CGAAGCCGACGAGAGAGTGT	/
	CTCTTATACGCGAGCACGGG	
	TCGGGTGCTGCACGTGATTG	
NF1	AACTTCGGAATTCTGCCTCT	Regulation of cell proliferation; regulation of cell death; regulation of transferase activity; regulation of MAPK cascade; regulation of cell differentiation; epithelium development; regulation of GTPase activity
	ACGGCCTGGACCCATTCCAC	
	GAGAGAAAATAAAACCCCAG	
NF2	GTCCATGGTGACGATCCTCA	Regulation of cell proliferation; regulation of transferase activity; regulation of MAPK cascade; regulation of cell differentiation; epithelium development
	GATCCTCACGGTGAACGTCT	
	ATCCTCACGGTGAACGTCTT	
NGFR	CGACGGCACGTATTCCGACG	Regulation of MAPK cascade
	CTCGTCGGAATACGTGCCGT	
	GCAGCGGCACACGGCGTCGT	
P2RY8	CTGGCGCCGCCGTCGTTACG	/
	GCGCCGCCGTCGTTACGCGG	
	CGGCCACCGCGTAACGACGG	
PAK7	CTACACGACCGAAAAGTACA	Regulation of cell death
	TTGAACACAGGGTTCATACT	
	ATGCATCACACCCATCCAGC	

Table S4 Continued

Gene	sgRNA	Function category
PARP3	ACCGTCATCCCGCACAACTT	/
	GTACACACTTATCGAAGTAC	
	TCGATAAGTGTGTACTTGCC	
PCDH7	TCACGGTGACCATCCCCACA	/
	ACCGACTGGCCGAACATGGG	
	GTACCGGAGGAGCTGCTTGG	
PDCD10	CACGGAGTCCCTTCTTCGTA	Regulation of cell proliferation; regulation of cell death; regulation of transferase activity; regulation of MAPK cascade; epithelium development
	ATAGAGGGGCATAGAAACCA	
	CATCAGCTGCCATACGAAGA	
PLK1	AATTTGCCGTAGGTAGTATC	Regulation of transcription from RNA polymerase II promoter; regulation of cell death; regulation of transferase activity
	AGCCAAGCACAATTTGCCGT	
	TACCTACGGCAAATTGTGCT	
PODN	CAGGAGGGCGTCGTGGACTG	Regulation of cell proliferation; regulation of transferase activity
	GCCCCAGGATTTGGCCGAAG	
	GCACCTGTACAACAACGCGC	
PRKCE	TCTTAAGATCAAAATCTGCG	Regulation of MAPK cascade
	CGTCAGGCGCAGGGTCCATC	
	AACGCATGCGGCCGAGGAAG	
PRKRA	ATTCAGGTATTACACGAATA	Regulation of cell proliferation; regulation of cell death
	TATTCGTGTAATACCTGAAT	
	TTCACCTTCAGAGTAACCGT	
PRPF4B	GAAAGTAGAAGTCGCGATCG	/
	GGAGCAGATCACGCTTGCGA	
	CACGCGGTGGTCGTAGACGA	
Pygo1	TATGGATTTGACGAAGGTAG	Regulation of transcription from RNA polymerase II promoter;
	AGATGGTCAGAGTTTGGATT	
	GACGAATGGCCATGACGGTT	
RANBP9	GTGAACATGAATAGACTACC	Regulation of MAPK cascade
	CTAATTCTTCTAGAACGGTC	
	GGCAGGACGACTCCGGAGAC	
RAP1GAP2	CTCGACCTGCACGACGATGT	Regulation of cell differentiation; regulation of GTPase activity
	ACAAAGCTGCCATTTACCGA	
	GATCCTGTCCGTCAAGTGCG	
RAPGEF1	TGAAGCCGTATACCTCCATG	Regulation of cell proliferation; regulation of transferase activity; regulation of MAPK cascade; regulation of cell differentiation; epithelium development; regulation of GTPase activity
	GAGATGTTGTCATACTGCGA	
	GCTCCCCCTGACAGACCGCG	

Table S4 Continued

Gene	sgRNA	Function category
RASSF5	AGTCCAACTGGATCAGGCTG	Regulation of cell proliferation
	GGATCAGGCTGCGGCATTCT	
	TGGATCAGGCTGCGGCATTC	
RBL1	GTACTGTGTGAACTGCATGA	Regulation of transcription from RNA polymerase II promoter; regulation of transferase activity
	AAATATCTCCGTCCGGTCAG	
	CCGCAAAAGCATTATTCCCA	
RHOG	TGACACCCTACGGCGCCTCA	Regulation of cell proliferation; regulation of GTPase activity
	GCGCCCATCACACCGCAGCA	
	GCGCGCAGAGCGCAGTTGAC	
ROCK1	GTAGTGAAGGTGATTGGTAG	Regulation of cell death; regulation of cell differentiation; epithelium development
	GTGATTGGTAGAGGTGCATT	
	TATGAAGTAGTGAAGGTGAT	
SPRED1	TATCCGTGGAGAGCGACTCA	Regulation of transferase activity; regulation of MAPK cascade
	TTATCCGTGGAGAGCGACTC	
	GGTGGATGGTTACCACTTGG	
TACC1	ACAGACCCAGTGGCACGAGA	/
	TCATGTGGTCAGAAATCAGC	
	TCAGCTGGTGCCGAGGTGAA	
TADA1	AATCTGACAACGCGTGAGAA	Covalent chromatin modification
	GACAACGCGTGAGAATGGCC	
	ACTGGGCTAACCTAAAGCTG	
TADA2B	GTAGCTAGGAAGGCGGCTTT	Regulation of transcription from RNA polymerase II promoter; covalent chromatin modification
	CGAGCTGAAGCGCGCCCACG	
	AACCTAGCCGGCTCCAAACG	
TAF5L	CAAACCGTTGTTCTCACTGC	Regulation of transcription from RNA polymerase II promoter; covalent chromatin modification
	CACGTCAAGATGAATATGTA	
	AAACAGTCCGAAGAGCACAG	
TAF6L	CCGCTCTTCTCGCTCTGACA	Regulation of transcription from RNA polymerase II promoter; covalent chromatin modification
	TGACGGTTGAGGACTTCAAC	
	TGAGCCGGACAGACTCCCGA	
TCEB2	AGCGGCCTCCTGACGAGCAG	Regulation of transcription from RNA polymerase II promoter;
	CGAACTGAAGCGCATCGTCG	
	GCTCGTCAGGAGGCCGCTTG	
TFAP2C	TTAAATGCCTCGTTACTGGG	Regulation of transcription from RNA polymerase II promoter; regulation of cell proliferation
	CTGTCTGATCGTGCAGCAAC	
	GAGGCATTTAAGCATTCAGG	

Table S4 Continued

Gene	sgRNA	Function category
TNFRSF1B	CACCCGGAGTATGGCCCCAG	Regulation of cell proliferation; regulation of cell death; regulation of MAPK cascade
	CGATAAGGCCCGGGGTACAC	
	ACTTGCCTGCCGATAAGGCC	
TNRC18	TATAAGGCTCTGCATGGCCG	/
	CCCGGCGCCGCCGTTGCACA	
	GAGCGGAAAGACAGGTCAGA	
TRIM67	ACAAGCTGAGCTTGTCCGCG	Regulation of cell differentiation
	GGCAGCACGCGAACCCCGTT	
	CAGGCAGTACTCCATCAGTC	
UBE2M	TGATATCACACGTCTTGGGC	Regulation of cell death
	TGAGGCAGACGTTGCCCTCG	
	GCGCAGCTGCGGATCCAGAA	
VAX1	GCCTTCCTCAAGGAGCCGCA	Regulation of transcription from RNA polymerase II promoter; regulation of cell proliferation; regulation of cell differentiation
	CTCGGACGCCGAGGCTGCCC	
	CAGCCGATAGAGCTGCTCCG	
YAP1	GACGTTCATCTGGGACAGCA	Regulation of transcription from RNA polymerase II promoter; regulation of cell proliferation; regulation of cell death; regulation of cell differentiation; epithelium development
	GGGGGCTGTGACGTTCATCT	
	TGGGGGCTGTGACGTTCATC	
ZNF182	ATTCTTCTACCTCCAACTTG	/
	CTACATGCAGTACATTCAAA	
	CCACACTCTTTATAGCCATA	
Negative control	CCTCGTTCACCGCCGTCGCG	/
	ATTTGCCGTTCGAATGCGTA	
	AAAGATAGGCCCTTCGCGGA	
	TCGACCGTAAAACCGGATAT	
	AAACTCACATGCTTGCGATC	
	ATACGCAAGCGTACCGACAA	
	TCGAAGGGTAACGAATCTA	
	AGTCCAAGGCGTTCGCGAAG	
	CCGTCAACGACGGCAAGCTG	
	TCATTGACGAATACCTCGAG	
	ACCGCGATAATCCTACAATT	
	AACGTCGACAGCGGGTACAC	

Table S5 List of the top 10% protective/sensitizing gene pairs

Protective gene pair	ρ score in replicate 1	*P* value in replicate 1	Protective gene pair	ρ score in replicate 1	*P* value in replicate 1
NF2 SPRED1	1.2378	1.58E-07	BCAR3 NF1	0.5706	6.38E-04
NF1 NF2	1.2333	2.69E-06	CUL3 TADA1	0.5632	8.58E-04
EZH2 NF2	1.0410	8.67E-06	CUL3 EAPP	0.5616	1.06E-03
CCNC NF2	1.0027	6.34E-06	RANBP9 SPRED1	0.5597	5.41E-04
NF2 TADA1	1.0023	8.82E-07	PCDH7 TAF5L	0.5566	6.38E-04
NF2 PCDH7	0.9780	4.59E-07	BCAR3 SPRED1	0.5564	1.94E-04
BCAR3 NF2	0.9682	5.36E-07	ARHGAP6 NF2	0.5533	4.17E-03
ARHGEF2 NF2	0.9222	4.14E-06	PARP3 RANBP9	0.5525	1.27E-03
MED12 NF2	0.9218	8.09E-06	ARHGAP6 BCAR3	0.5509	1.18E-03
NF2 TADA2B	0.9129	2.16E-06	MAP3K11 NF2	0.5473	5.26E-03
CUL3 NF2	0.8864	1.50E-05	NF2 PLK1	0.5468	1.69E-03
LPAR5 NF2	0.8787	2.32E-06	LPAR5 PCDH7	0.5391	7.92E-04
NF2 TAF5L	0.8658	2.59E-06	PCDH7 BIRC5	0.5362	7.71E-04
METTL21B NF2	0.8642	2.79E-06	BCAR3 EED	0.5343	6.74E-04
KDM5B NF2	0.8597	1.03E-05	NF1 RASSF5	0.5314	1.82E-03
EPAS1 NF2	0.8586	3.11E-05	NF2 P2RY8	0.5298	1.53E-03
NF2 TRIM67	0.8536	7.54E-06	DRAP1 PCDH7	0.5297	9.55E-04
KRAS NF2	0.8533	3.00E-06	EGFR NF1	0.5255	4.58E-03
NF2 TACC1	0.8426	8.37E-06	CUL3 TAF6L	0.5233	8.58E-04
NF2 PDCD10	0.8165	5.19E-05	NF2 UBE2M	0.5207	2.78E-03
EED NF2	0.8109	2.64E-05	MECOM NF2	0.5196	1.87E-03
DCLK1 NF2	0.8064	1.96E-05	RANBP9 RAP1GAP2	0.5196	1.27E-03
NF2 Pygo1	0.8013	2.82E-05	EphA2 NF2	0.5176	1.01E-03
NF2 TFAP2C	0.8003	4.78E-06	NF1 TFAP2C	0.5157	9.81E-04
EGFR NF2	0.7979	2.64E-05	NF2 RAP1GAP2	0.5155	2.10E-02
NF1 TAF5L	0.7859	1.31E-05	NF2 RBL1	0.5148	2.28E-03
NF2 ROCK1	0.7850	2.24E-05	NF1 TADA2B	0.5096	2.23E-03
NF2 PAK7	0.7783	7.29E-06	NF1 UBE2M	0.5084	3.53E-03
MED15 NF2	0.7782	4.57E-05	EphA2 PCDH7	0.5077	7.71E-04
EAPP NF2	0.7743	1.50E-05	DRAP1 NF2	0.5071	9.39E-03
MRFAP1L1 NF2	0.7709	1.35E-04	FSD1 NF1	0.4962	1.92E-03
BAP1 NF2	0.7679	1.22E-05	KRAS NF1	0.4946	5.96E-02
HMMR NF2	0.7673	1.66E-05	C9orf50 NF2	0.4942	6.31E-03
GPR35 NF2	0.7671	1.22E-05	CUL3 NF1	0.4926	2.60E-02
GAB2 NF2	0.7626	1.66E-05	CCDC101 RANBP9	0.4885	2.46E-03

Table S5 Continued

Protective gene pair	ρ score in replicate 1	*P* value in replicate 1	Protective gene pair	ρ score in replicate 1	*P* value in replicate 1
NF2 YAP1	0.7620	2.82E-05	CCDC101 PCDH7	0.4880	2.07E-03
CHN2 NF2	0.7589	2.64E-05	BCAR3 ZNF182	0.4866	3.53E-03
CUL3 EZH2	0.7586	1.40E-05	CCNC RANBP9	0.4837	5.38E-03
CCDC101 NF2	0.7494	2.91E-05	BCAR3 TADA1	0.4834	2.99E-03
NF2 RASSF5	0.7480	4.87E-05	CUL3 LGR5	0.4828	2.40E-03
NF2 ZNF182	0.7426	2.91E-05	NF1 RANBP9	0.4812	2.17E-03
NF2 BIRC5	0.7422	1.22E-05	BCAR3 RANBP9	0.4760	4.27E-03
NF2 PODN	0.7410	1.44E-04	CUL3 UBE2M	0.4745	2.40E-03
CRB2 NF2	0.7384	5.53E-05	NF2 TNFRSF1B	0.4701	5.76E-03
HDGF NF2	0.7367	5.90E-05	MRFAP1L1 PCDH7	0.4677	6.03E-03
NF2 VAX1	0.7363	2.82E-05	BCAR3 GAB2	0.4622	3.62E-03
LAMA3 NF2	0.7262	2.12E-04	MED12 NF1	0.4532	5.90E-03
LCOR NF2	0.7260	1.96E-05	NF1 TACC1	0.4487	4.47E-03
MAPKAPK3 NF2	0.7246	3.54E-05	NF1 RHOG	0.4452	9.60E-03
FSD1 NF2	0.7237	8.06E-05	HDAC6 NF2	0.4410	1.56E-02
NGFR NF2	0.7147	4.87E-05	CUL3 SPRED1	0.4387	2.65E-02
NF1 PCDH7	0.7111	3.77E-05	BCAR3 Pygo1	0.4369	8.80E-03
HOXC13 NF2	0.7058	6.74E-04	BCAR3 CCDC101	0.4369	5.51E-03
NF2 TNRC18	0.7053	3.46E-04	PCDH7 RANBP9	0.4354	8.99E-03
NF2 TAF6L	0.7039	1.53E-04	CUL3 PARP3	0.4352	1.38E-02
NF2 RAPGEF1	0.7032	3.21E-05	BCAR3 UBE2M	0.4344	7.22E-03
NF1 PDCD10	0.7027	3.01E-05	PCDH7 TACC1	0.4337	1.02E-02
IGF1R NF2	0.7003	2.24E-05	NF1 BIRC5	0.4325	2.92E-03
NF2 RANBP9	0.6955	2.12E-04	MECOM PCDH7	0.4302	2.81E-02
LGR5 NF2	0.6842	6.48E-05	PDCD10 RANBP9	0.4301	1.19E-02
ITGB3 NF2	0.6838	1.44E-04	SPRED1 TAF5L	0.4288	4.91E-03
NF2 RHOG	0.6808	4.72E-05	EPAS1 PCDH7	0.4287	9.81E-03
HMMR PCDH7	0.6772	8.06E-05	PCDH7 PODN	0.4270	1.11E-02
KDM5B RANBP9	0.6756	2.55E-05	APBB1 NF2	0.4257	3.71E-02
CUL3 PCDH7	0.6748	1.88E-04	ARHGEF5 NF2	0.4224	6.90E-03
BCAR3 PCDH7	0.6709	5.36E-05	C9orf50 PCDH7	0.4170	1.14E-02
NF2 PRPF4B	0.6622	1.77E-04	ARHGAP6 TADA1	0.4153	1.24E-02
NF2 PRKRA	0.6520	3.00E-04	BAP1 NF1	0.4139	4.47E-03
ADI1 NF2	0.6463	5.88E-04	CUL3 Pygo1	0.4106	4.52E-02
CUL3 ZNF182	0.6426	2.78E-03	P2RY8 RANBP9	0.4088	2.65E-02

Table S5 Continued

Protective gene pair	ρ score in replicate 1	*P* value in replicate 1	Protective gene pair	ρ score in replicate 1	*P* value in replicate 1
BCAR3 CUL3	0.6377	2.24E-04	CUL3 KDM5B	0.4088	2.19E-02
Jun NF2	0.6338	1.28E-04	BCAR3 BIRC5	0.4059	8.42E-03
ADI1 ARHGAP6	0.6171	8.84E-05	BCAR3 TAF5L	0.3987	1.09E-02
ARHGAP6 PCDH7	0.6092	3.45E-04	HOXC13 RANBP9	0.3961	4.36E-02
PCDH7 TNRC18	0.6080	1.24E-04	BCAR3 PDCD10	0.3957	1.49E-02
ADI1 RANBP9	0.6041	6.04E-04	PCDH7 PRPF4B	0.3922	1.87E-02
PCDH7 ZNF182	0.5942	2.38E-04	PCDH7 UBE2M	0.3908	2.23E-02
GPR98 NF2	0.5734	4.45E-04	NF2 PARP3	0.3870	6.59E-02
EZH2 NF1	0.5718	1.24E-02	CCNC PCDH7	0.3844	1.65E-02
CUL3 RANBP9	0.5716	2.18E-04			

Sensitizing gene pair	ρ score in replicate 1	*P* value in replicate 1	Sensitizing gene pair	ρ score in replicate 1	*P* value in replicate 1
PRPF4B RAP1GAP2	−0.8391	3.54E-05	HOXC13 MECOM	−0.5028	2.17E-03
GBP2 LAMA3	−0.7970	5.53E-05	IGF1R PARP3	−0.5015	7.71E-03
CRB2 RBL1	−0.7964	1.34E-03	EAPP METTL21B	−0.5009	1.38E-03
ARHGEF2 C9orf50	−0.7725	9.70E-05	ARHGEF5 TNFRSF1B	−0.5008	4.69E-03
RAP1GAP2 RBL1	−0.7538	3.86E-06	EphA2 METTL21B	−0.4974	1.40E-02
EGFR PLK1	−0.7528	1.28E-04	ITGB3 RAP1GAP2	−0.4944	1.07E-02
GPR98 TAF6L	−0.7287	2.67E-04	ARHGEF2 MED15	−0.4907	7.06E-03
NGFR MED15	−0.6944	7.71E-04	C9orf50 NGFR	−0.4840	3.71E-02
BUB1B ITGB3	−0.6828	3.62E-03	GBP2 MAP3K11	−0.4827	3.14E-02
PRPF4B RHOG	−0.6477	4.76E-02	ARHGEF5 GBP2	−0.4821	1.82E-03
HDGF LGR5	−0.6397	3.37E-03	EAPP RHOG	−0.4770	5.63E-03
NGFR PRPF4B	−0.6255	2.85E-03	CHN2 PODN	−0.4701	5.38E-03
PARP3 VAX1	−0.6236	1.34E-03	HDAC6 PODN	−0.4696	1.38E-02
PARP3 PODN	−0.6228	6.31E-03	PRPF4B VAX1	−0.4687	1.14E-02
C9orf50 MED15	−0.6219	1.57E-03	MAPKAPK3 VAX1	−0.4671	6.17E-03
ADI1 P2RY8	−0.5932	6.38E-04	GPR98 MRFAP1L1	−0.4658	1.50E-01
NGFR MED12	−0.5902	1.27E-02	HMMR RAP1GAP2	−0.4657	4.93E-02
PRPF4B TNRC18	−0.5850	2.12E-03	ITGB3 PRKRA	−0.4642	6.31E-03
C9orf50 ITGB3	−0.5840	4.27E-03	Pygo1 RAP1GAP2	−0.4624	4.17E-03
EAPP TAF6L	−0.5825	2.71E-03	ITGB3 TNRC18	−0.4619	2.23E-02
IGF1R ITGB3	−0.5796	3.45E-03	GPR98 IGF1R	−0.4571	7.27E-02
LAMA3 RAP1GAP2	−0.5737	3.21E-03	GBP2 GPR35	−0.4570	2.14E-02

Table S5 Continued

Sensitizing gene pair	ρ score in replicate 1	*P* value in replicate 1	Sensitizing gene pair	ρ score in replicate 1	*P* value in replicate 1
BUB1B C9orf50	−0.5692	1.18E-03	PLK1 PRKRA	−0.4488	8.24E-03
MECOM RBL1	−0.5596	1.27E-02	MAP3K11 PRPF4B	−0.4479	1.98E-02
IGF1R PRPF4B	−0.5593	2.85E-03	APBB1 ITGB3	−0.4449	9.60E-03
ARHGEF2 KRAS	−0.5537	3.85E-02	ARHGEF2 Jun	−0.4441	2.02E-02
ITGB3 Jun	−0.5513	2.86E-02	ARHGEF2 LCOR	−0.4431	1.40E-02
ARHGEF5 RAP1GAP2	−0.5479	1.92E-03	GPR98 PLK1	−0.4390	2.85E-03
ADI1 PRKCE	−0.5336	8.61E-03	C9orf50 RAP1GAP2	−0.4328	9.38E-02
METTL21B PODN	−0.5328	1.49E-02	METTL21B RAP1GAP2	−0.4308	1.25E-01
MED15 RAPGEF1	−0.5282	1.07E-02	ADI1 GBP2	−0.4289	1.87E-02
ARHGEF5 KRAS	−0.5193	3.29E-03	GPR98 MED15	−0.4246	9.19E-03
GBP2 MECOM	−0.5170	1.19E-02	PRPF4B RBL1	−0.4229	1.79E-02
MED15 TNRC18	−0.5083	2.41E-02	MED12 VAX1	−0.4089	1.65E-02

Table S6 List of strongly interacted protective/sensitizing gene pairs (data shown below were calculated using sample Replicate 1)

Strongly interacted protective gene pair (A–B)	Score of gene pair	Score of gene A	Score of gene B	*P* value	*GI* score (Linear fitting)	*GI* score (Loewe's additivity)	Interaction type
ADI1 ARHGAP6	0.6171	−0.1868	−0.0117	8.84E-05	0.559	0.4186	Synergistic
BUB1B RAPGEF1	0.4	−0.2735	−0.1869	3.64E-02	0.4882	−0.0604	Antagonistic
BUB1B YAP1	0.346	−0.2735	−0.2184	3.67E-01	0.4711	−0.1459	Antagonistic
BUB1B EAPP	0.263	−0.2735	−0.4878	1.44E-01	0.4691	−0.4983	Antagonistic
RANBP9 RAP1GAP2	0.5196	0.1274	−0.5324	1.27E-03	0.4677	0.1146	Synergistic
ARHGEF2 CCNC	0.2584	−0.3823	−0.3631	1.77E-01	0.4634	−0.487	Antagonistic
PARP3 RANBP9	0.5525	−0.6766	0.1274	1.27E-03	0.4601	0.0033	Synergistic
ARHGAP6 CRB2	0.4747	−0.0117	−0.346	3.71E-03	0.4214	0.117	Synergistic t
NF2 SPRED1	1.2378	0.6859	0.2327	1.58E-07	0.4191	0.3192	Synergistic
ADI1 RANBP9	0.6041	−0.1868	0.1274	6.04E-04	0.4128	0.5447	Synergistic
NF1 NF2	1.2333	0.3664	0.6859	2.69E-06	0.4112	0.181	Synergistic
HDAC6 ITGB3	0.2818	−0.0889	−0.2108	1.06E-01	0.4086	−0.0179	Antagonistic
IGF1R KRAS	0.2178	−0.2905	−0.1942	2.27E-01	0.4083	−0.2669	Antagonistic
ARHGEF2 MAP3K11	0.1864	−0.3823	−0.2784	2.45E-01	0.4071	−0.4743	Antagonistic
LAMA3 PRKRA	0.4808	−0.1123	0.0484	3.13E-03	0.3989	0.4169	Synergistic
EED PRKRA	0.4796	−0.2929	0.0484	6.03E-03	0.3794	0.2351	Synergistic
HOXC13 RAP1GAP2	0.1022	−0.1916	−0.5324	3.78E-01	0.3702	−0.6218	Antagonistic
NF1 PDCD10	0.7027	0.3664	−0.2751	3.01E-05	0.3661	0.6114	Synergistic
HOXC13 RAPGEF1	0.3506	−0.1916	−0.1869	6.92E-02	0.3655	−0.0279	Antagonistic

Table S6 Continued

Strongly interacted protective gene pair (A–B)	Score of gene pair	Score of gene A	Score of gene B	*P* value	*GI* score (Linear fitting)	*GI* score (Loewe's additivity)	Interaction type
CCNC NF2	1.0027	−0.3631	0.6859	6.34E-06	0.3607	0.6799	Synergistic
CUL3 EZH2	0.7586	0.237	0.1242	1.40E-05	0.3577	0.3974	Synergistic
ARHGAP6 PARP3	0.2903	−0.0117	−0.6766	1.19E-01	0.356	−0.398	Antagonistic
ITGB3 TNFRSF1B	0.0462	−0.2108	−0.4666	7.41E-01	0.3543	−0.6312	Antagonistic
EZH2 PARP3	0.3828	0.1242	−0.6766	2.65E-02	0.3521	−0.1696	Antagonistic
GPR98 Pygo1	0.0055	−0.4642	−0.245	7.58E-01	0.3507	−0.7037	Antagonistic
HDAC6 HOXC13	0.3195	−0.0889	−0.1916	5.76E-02	0.3427	0.039	Synergistic
PRKCE UBE2M	0.2688	−0.1565	−0.1777	1.63E-01	0.3346	−0.0654	Antagonistic
ARHGEF2 TCEB2	0.26	−0.3823	0.0444	2.33E-01	0.333	−0.0779	Antagonistic
GBP2 HMMR	0.2354	−0.1525	−0.1555	1.23E-01	0.3314	−0.0726	Antagonistic
ADI1 APBB1	0.2406	−0.1868	−0.2178	8.40E-02	0.3205	−0.164	Antagonistic
P2RY8 RAPGEF1	0.2076	−0.2296	−0.1869	5.55E-01	0.3182	−0.2089	Antagonistic
EPAS1 EphA2	0.3336	−0.1449	0.0731	2.36E-02	0.3111	0.2618	Synergistic
CCNC UBE2M	0.2417	−0.3631	−0.1777	3.40E-01	0.3094	−0.2991	Antagonistic
PRKCE RAP1GAP2	−0.0144	−0.1565	−0.5324	7.92E-01	0.3067	−0.6745	Antagonistic
PLK1 TAF6L	0.1553	−0.2568	−0.1632	3.00E-01	0.3066	−0.2647	Antagonistic
EGFR NF2	0.7979	−0.1807	0.6859	2.64E-05	0.3011	0.2927	Synergistic
NGFR RAP1GAP2	−0.0859	−0.2402	−0.5324	4.33E-01	0.2988	−0.6867	Antagonistic
GPR98 HOXC13	0.0585	−0.4642	−0.1916	9.32E-01	0.294	−0.5973	Antagonistic
MED12 NF2	0.9218	−0.0491	0.6859	8.09E-06	0.2897	0.285	Synergistic
APBB1 RBL1	0.1863	−0.2178	−0.1266	3.83E-01	0.2729	−0.1581	Antagonistic
TFAP2C UBE2M	0.2464	−0.053	−0.1777	1.01E-01	0.2729	0.0157	Synergistic
EAPP HOXC13	0.1284	−0.4878	−0.1916	1.55E-01	0.2637	−0.551	Antagonistic
CCNC RANBP9	0.4837	−0.3631	0.1274	5.38E-03	0.2589	0.248	Synergistic

Strongly interacted sensitizing gene pair (A–B)	Score of gene pair	Score of gene A	Score of gene	*P* value	*GI* score (Linear fitting)	*GI* score (Loewe's additivity)	Interaction type
GBP2 LAMA3	−0.797	−0.1525	−0.1123	5.53E-05	−0.6777	0.5322	Synergistic
EGFR PLK1	−0.7528	−0.1807	−0.2568	1.28E-04	−0.6345	0.3153	Synergistic
GPR98 ZNF182	−0.7592	−0.4642	−0.0667	2.24E-04	−0.5901	0.2283	Synergistic
HDGF LGR5	−0.6397	−0.0575	−0.2154	3.37E-03	−0.5871	0.3668	Synergistic
NGFR MED15	−0.6944	−0.2402	−0.0826	7.71E-04	−0.5732	0.3716	Synergistic
LPAR5 PARP3	−1.0143	−0.306	−0.6766	8.37E-06	−0.5652	0.0317	Synergistic
NGFR MED12	−0.5902	−0.2402	−0.0491	1.27E-02	−0.5631	0.3009	Synergistic
CUL3 PODN	−0.3459	0.237	−0.0846	5.47E-02	−0.5486	0.1935	Synergistic

Table S6 Continued

Strongly interacted sensitizing gene pair (A–B)	Score of gene pair	Score of gene A	Score of gene	*P* value	*GI* score (Linear fitting)	*GI* score (Loewe's additivity)	Interaction type
PRPF4B TFAP2C	−0.7627	−0.3752	−0.053	2.23E-03	−0.5335	0.3345	Synergistic
PRKRA YAP1	−0.4496	0.0484	−0.2184	4.68E-02	−0.5188	0.2796	Synergistic
MED15 TNRC18	−0.5083	−0.0826	−0.0969	2.41E-02	−0.5025	0.3288	Synergistic
MED15 RAPGEF1	−0.5282	−0.0826	−0.1869	1.07E-02	−0.5022	0.2587	Synergistic
BUB1B ITGB3	−0.6828	−0.2735	−0.2108	3.62E-03	−0.4725	0.1985	Synergistic
GPR98 TAF6L	−0.7287	−0.4642	−0.1632	2.67E-04	−0.4468	0.1013	Synergistic
METTL21B PODN	−0.5328	−0.0836	−0.0846	1.49E-02	−0.4412	0.3646	Synergistic
PLK1 PRKRA	−0.4488	−0.2568	0.0484	8.24E-03	−0.4334	0.2404	Synergistic
ITGB3 PRKRA	−0.4642	−0.2108	0.0484	6.31E-03	−0.4304	0.3018	Synergistic
HDAC6 PODN	−0.4696	−0.0889	−0.0846	1.38E-02	−0.422	0.2961	Synergistic
ADI1 P2RY8	−0.5932	−0.1868	−0.2296	6.38E-04	−0.4139	0.1768	Synergistic
ADI1 PRKCE	−0.5336	−0.1868	−0.1565	8.61E-03	−0.4134	0.1903	Synergistic
C9orf50 MED15	−0.6219	−0.3004	−0.0826	1.57E-03	−0.4089	0.2389	Synergistic
BAP1 EGFR	−0.4587	−0.2036	−0.1807	8.99E-03	−0.4016	0.0744	Synergistic
RAP1GAP2 RBL1	−0.7538	−0.5324	−0.1266	3.86E-06	−0.3989	0.0948	Synergistic
MED12 TFAP2C	−0.3342	−0.0491	−0.053	1.79E-02	−0.393	0.2321	Synergistic
EAPP TAF6L	−0.5825	−0.4878	−0.1632	2.71E-03	−0.3919	−0.0685	Antagonistic
KRAS P2RY8	−0.5754	−0.1942	−0.2296	2.02E-02	−0.3917	0.1516	Synergistic
ITGB3 Jun	−0.5513	−0.2108	−0.166	2.86E-02	−0.391	0.1745	Synergistic
MECOM RBL1	−0.5596	−0.2327	−0.1266	1.27E-02	−0.3875	0.2003	Synergistic
MED12 VAX1	−0.4089	−0.0491	−0.3051	1.65E-02	−0.3772	0.0547	Synergistic
GBP2 PRKRA	−0.3831	−0.1525	0.0484	1.29E-02	−0.3564	0.279	Synergistic
PRPF4B TNRC18	−0.585	−0.3752	−0.0969	2.12E-03	−0.354	0.1129	Synergistic
HDAC6 YAP1	−0.3852	−0.0889	−0.2184	1.92E-01	−0.3535	0.0779	Synergistic
IGF1R ITGB3	−0.5796	−0.2905	−0.2108	3.45E-03	−0.342	0.0783	Synergistic
ITGB3 TNRC18	−0.4619	−0.2108	−0.0969	2.23E-02	−0.3286	0.1542	Synergistic
GBP2 MECOM	−0.517	−0.1525	−0.2327	1.19E-02	−0.3196	0.1318	Synergistic
ARHGEF2 MED15	−0.4907	−0.3823	−0.0826	7.06E-03	−0.3191	0.0258	Synergistic
APBB1 ITGB3	−0.4449	−0.2178	−0.2108	9.60E-03	−0.3147	0.0163	Synergistic
DCLK1 EZH2	−0.0324	0.0796	0.1242	5.45E-01	−0.3146	−0.1714	Antagonistic
CHN2 PODN	−0.4701	−0.3555	−0.0846	5.38E-03	−0.3136	0.03	Synergistic
ARHGEF5 KRAS	−0.5193	−0.2593	−0.1942	3.29E-03	−0.3128	0.0658	Synergistic
PARP3 PODN	−0.6228	−0.6766	−0.0846	6.31E-03	−0.3117	−0.1384	Antagonistic
PRPF4B RAP1GAP2	−0.8391	−0.3752	−0.5324	3.54E-05	−0.3074	−0.0685	Antagonistic
ITGB3 TAF6L	−0.4514	−0.2108	−0.1632	4.06E-02	−0.3057	0.0774	Synergistic

Table S6 Continued

Strongly interacted sensitizing gene pair (A–B)	Score of gene pair	Score of gene A	Score of gene	*P* value	*GI* score (Linear fitting)	*GI* score (Loewe's additivity)	Interaction type
EphA2 MRFAP1L	−0.2778	0.0731	−0.1608	4.13E-02	−0.3022	0.1901	Synergistic
GBP2 MAP3K11	−0.4827	−0.1525	−0.2784	3.14E-02	−0.3009	0.0518	Synergistic
NGFR PRPF4B	−0.6255	−0.2402	−0.3752	2.85E-03	−0.3	0.0101	Synergistic
RAPGEF1 TFAP2C	−0.3292	−0.1869	−0.053	4.93E-02	−0.2991	0.0893	Synergistic
LAMA3 MAPKAPK3	−0.3801	−0.1123	−0.1867	1.87E-02	−0.299	0.0811	Synergistic
LAMA3 PODN	−0.3477	−0.1123	−0.0846	7.15E-02	−0.2938	0.1508	Synergistic
EZH2 METTL21B	−0.1172	0.1242	−0.0836	9.14E-01	−0.2867	0.0766	Synergistic
APBB1 NGFR	−0.3974	−0.2178	−0.2402	1.72E-02	−0.2836	−0.0606	Antagonistic
MAPKAPK3 VAX1	−0.4671	−0.1867	−0.3051	6.17E-03	−0.2814	−0.0247	Antagonistic
EAPP METTL21B	−0.5009	−0.4878	−0.0836	1.38E-03	−0.2799	−0.0705	Antagonistic
PRPF4B RHOG	−0.6477	−0.3752	−0.3318	4.76E-02	−0.276	−0.0593	Antagonistic
BAP1 CUL3	−0.0696	−0.2036	0.237	4.82E-01	−0.2738	0.0362	Synergistic
GBP2 YAP1	−0.3912	−0.1525	−0.2184	4.13E-02	−0.2681	0.0203	Synergistic
ADI1 GBP2	−0.4289	−0.1868	−0.1525	1.87E-02	−0.268	0.0896	Synergistic
BUB1B C9orf50	−0.5692	−0.2735	−0.3004	1.18E-03	−0.2676	−0.0047	Antagonistic
GBP2 GPR35	−0.457	−0.1525	−0.1666	2.14E-02	−0.2675	0.1379	Synergistic

Table S7 Information of siRNAs used in the three-dimensional spheroid formation assay and subsequent mechanism dissection

Item	Sequence
siLGR5-forward	CUCUGAUGAUGUCGAAAAATT
siLGR5-reverse	UUUUUCGACAUCAUCAGAGTT
siHDGF-forward	CUAUGGAGGUGGAAAAGAATT
siHDGF-reverse	UUCUUUUCCACCUCCAUAGTT
